# Adipose-Derived Stem Cells in Novel Approaches to Breast Reconstruction: Their Suitability for Tissue Engineering and Oncological Safety

**DOI:** 10.1177/1178223417726777

**Published:** 2017-08-16

**Authors:** Niamh O’Halloran, Donald Courtney, Michael J Kerin, Aoife J Lowery

**Affiliations:** 1 Discipline of Surgery, Lambe Institute for Translational Research, National University of Ireland, Galway, Galway, Ireland; 2 Graduate Entry Medical School, University of Limerick, Limerick, Ireland

**Keywords:** Breast reconstruction, mastectomy, adipose-derived stem cells, tissue engineering, adipose tissue engineering, breast cancer

## Abstract

Adipose-derived stem cells (ADSCs) are rapidly becoming the gold standard cell source for tissue engineering strategies and hold great potential for novel breast reconstruction strategies. However, their use in patients with breast cancer is controversial and their oncological safety, particularly in relation to local disease recurrence, has been questioned. In vitro, in vivo, and clinical studies using ADSCs report conflicting data on their suitability for adipose tissue regeneration in patients with cancer. This review aims to provide an overview of the potential role for ADSCs in breast reconstruction and to examine the evidence relating to the oncologic safety of their use in patients with breast cancer.

## Introduction

Breast cancer represents a significant health care burden, with an estimated 1.7 million new cases diagnosed worldwide annually.^1^ Approximately 40% of patients with breast cancer require mastectomy to achieve locoregional disease control; recent trends show that higher numbers of women eligible for breast-conserving surgery (BCS) are opting for mastectomy and rates of contralateral and bilateral prophylactic mastectomy are also rising.^2–7^ Mastectomy is associated with significant aesthetic and psychosocial morbidity, which is improved by breast reconstruction.^8,9^ Current reconstructive approaches include autologous tissue transfer, prosthetic implants, and biological matrices; however, these approaches remain limited by the potential for complications at the donor and reconstruction sites. Increasing patient expectations for improved aesthetic outcomes means that surgeons are persistently attempting to optimise surgical technique and investigating new and improved approaches to breast reconstruction. This has driven research in the direction of tissue engineering strategies in an effort to develop superior breast reconstruction alternatives. Adipose-derived stem cells (ADSCs) have become the gold standard as a cell source for tissue engineering.^10^ They are particularly attractive for breast reconstruction as they exhibit potential for proliferation, preferential differentiation to adipocytes, and maintenance of mature adipose graft volume. However, the oncological safety of their use for adipose tissue regeneration, particularly in patients who have had a malignancy has been questioned.^11–15^ Concern stems from the characteristics that make ADSCs attractive for tissue engineering, namely, their proliferative and differentiation capacity along with stromal support of cancer cells and delivery of locally inflammatory cytokines and/or growth factors. The aim of this review is to examine the current use of ADSCs in adipose tissue engineering, specifically related to breast reconstruction with a focus on cellular biology; use in breast surgery; oncological safety; and the effect of adjuvant therapies on the regenerative potential of ADSCs.

## Breast Reconstruction: Current Approaches and Limitations

The National Institute of Clinical Excellence (NICE) guidelines recommend that all suitable patients undergoing mastectomy for breast cancer should be offered immediate breast reconstruction.^16^ Contemporary breast reconstruction approaches can be categorised as follows:

1. *Implant-based reconstruction.* Encompassing (a) implant-only reconstructions performed as a 2-stage procedure with placement of a tissue expander which is subsequently replaced with a permanent implant at a later operation or (b) single-stage, direct to fixed-volume permanent implant reconstruction with or without an acellular dermal matrix (ADM);
2. *Autologous reconstruction*. Using pedicled tissue flaps (eg, latissimus dorsi [LD] flap), which tend to be myocutaneous, or free tissue transfer (eg, deep inferior epigastric perforator [DIEP] flap), which may be composed of myocutaneous tissue or solely adipose tissue along with perforating vasculature.

Until recently, autologous flap procedures were the most common reconstructive approach. Contemporary trends have seen implant-based reconstruction become more common in the United States and Europe,^17–21^ possibly explained by shorter and less complicated procedures and increased use of ADMs which have been shown to improve cosmetic outcomes by allowing for better definition of the infra- and lateral mammary folds, reduced capsular contracture rates, and the provision of an additional biocompatible layer between the prosthesis and the overlying skin.^22^ Traditionally, single-stage immediate implant-based reconstruction with total muscle coverage of the implant was only achievable in small-breasted women as it is limited by the degree of expansion of the overlying pectoral muscles. This may be overcome by placement of a tissue expander prosthesis which can be inflated over time and replaced by a permanent implant at a second surgery, or alternatively using an ADM with the permanent implant, obviating the need for total muscle coverage.^23^ Short-term complications of implant-based reconstructions include seroma, haematoma, infection, and skin necrosis, with implant extrusion and rupture being long-term possibilities.^24,25^ A significant longer term complication of implants is capsular contracture; the formation of a firm, fibrous tissue capsule surrounding the implant. This constricts over time, resulting in a spherical appearance of the breast which feels firmer than desired, chronic chest wall discomfort, and restricted shoulder rotation. The increasing use of ADMs in implant-based reconstruction has reduced the rate of capsular contracture; however, it remains a significant problem. Capsular contracture has a cumulative incidence of 6% to 18% in non–ADM-assisted implant reconstructions, compared with <5% in ADM-assisted procedures.^26^ Post-mastectomy radiation therapy (PMRT) has deleterious effects on aesthetic outcomes and complication rates in implant-based reconstruction as it can affect the symmetry, volume, and projection initially achieved at the time of reconstruction. Post-mastectomy radiation therapy also increases the rates of grade 3 and 4 capsular contracture and reduces the skin quality of the mastectomy flaps^27^ leading to an increased risk of necrosis and implant loss.

Although the number of autologous reconstructions being performed has been surpassed by implant-based approaches, this approach still has a prominent role in post-mastectomy breast reconstruction, particularly in patients who have poor skin quality of the mastectomy flaps or for whom delayed reconstruction is preferred.^28^ The most widely used pedicled flap was traditionally the LD flap^29^; however, this is now being surpassed by the DIEP flap, although LD reconstruction is still popular as a salvage or delayed breast reconstruction technique.^30,31^ Free flaps include DIEP flaps and transverse rectus abdominis muscle (TRAM) flaps, which is also used as a pedicled flap. More recently developed flaps include the superior and inferior gluteal artery perforator flaps, transverse upper gracilis flap, superficial inferior epigastric artery flap, and profunda artery perforator flap. Autologous reconstructions are more cosmetically natural in shape and texture than implants. They provide skin coverage in cases of poor quality of the mastectomy flaps or delayed breast reconstruction. It is believed that DIEP reconstruction is more suitable in patients who will require PMRT as muscular atrophy is a significant complication of LD reconstruction that may occur post-radiotherapy.^32^

Although initial complication rates for autologous reconstructions may be higher, they provide a more consistent and durable reconstruction over time.^33^ Unfortunately, autologous reconstruction is associated with morbidity at the donor *and* reconstruction site. Tissue flap necrosis and loss may occur secondary to ischaemia of transferred tissue. Complications may arise from the donor site in the form of, eg, an incisional hernia in the case of a TRAM flap (incidence of 1.2%-8%) or donor site seroma in LD flaps (incidence of 70%-80%).^34,35^ These operations require longer admissions and recovery times.^36^ Autologous flap procedures are also longer and more technically challenging, particularly in the case of DIEP flaps which require the formation of a microvascular anastomosis.^37^

Due to the complications associated with current breast reconstruction methods, there is an urgent need to develop superior alternatives that will achieve the aesthetic goal of establishing a natural appearing breast shape. The preferred approach would include an autologous or biocompatible component to minimise foreign body reactions but without the requirement for extensive surgical resection at a donor site. Regenerative medicine approaches hold exciting potential in this regard, and recent efforts have focused on cell-based regeneration of adipose tissue.

## Adipose-Derived Stem Cells

There has been increasing interest the potential of autologous fat as a donor source for effective breast reconstruction. Autologous fat is thought to be a superior method of soft tissue augmentation due to a range of properties including biocompatibility and versatility; it is non-immunogenic, has similar mechanical properties to breast tissue, appears more natural than implants or pedicled flaps, and is associated with minimal donor site morbidity.^38^ Recent scientific interest has focused on the potential for adipose tissue engineering to generate sufficient volumes of fat for breast reconstruction. Adipose tissue engineering requires a stem cell with the capacity for differentiation into mature adipocytes.

Stem cells are an undifferentiated cell type with multipotent capacity.^39,40^ Adult/somatic stem cells are multipotent cells within adult tissues which maintain and repair the tissue in which they are found and are capable of differentiating into mature cell types such as osteoblasts, adipocytes, and chondroblasts, in addition to a lack of expression of HLA-DR surface molecules.^41^ Adult/somatic stem cells are more abundantly available and avoid the ethical considerations associated with the use of embryonic stem cells (ESCs) for tissue regeneration.^10,42^ Adult stem cells are found in almost all adult tissues; mesenchymal stem cells (MSCs) have been harvested from tissues such as trabecular bone and periosteum, synovial membrane, skeletal muscle, skin, teeth, and periodontal ligaments.^10,43–49^ However, the most widely harvested and studied adult stem cells are those from bone marrow, adipose tissue, and peripheral blood.^50^ Adipose-derived stem cells are rapidly becoming the gold standard as a cell source for tissue engineering and regenerative medicine. They are contained within the stromal vascular fraction (SVF) of adipose tissue and hypothesised to improve wound healing, tissue regeneration, and graft retention.^51^ According to the International Federation for Adipose Therapeutics and Science (IFATS) and International Society for Cellular Therapy (ISCT) joint statement on ADSCs, these cells are identified phenotypically as a CD45^−^, CD235a^−^, CD31^−^, and CD34^+^ cell population. They differ from bone marrow–derived cells (BMSCs) in that they are positive for CD36 and negative for CD106. They are also capable of trilineage differentiation.^52^

Adipose-derived stem cells possess certain advantages over BMSCs and ESCs. They are isolated with less invasive techniques, offer a higher cell yield than bone marrow aspirates (>1000× stem cell number per gram of tissue) or umbilical cord blood,^40,53^ have significant proliferative capacity in culture with a longer life span in culture than BMSCs,^10,54^ and possess multi-lineage potential (eg, adipogenic, osteogenic, myogenic, cardiomyogenic, and neurogenic cell types).^55–58^ Adipose-derived stem cells also have a shorter doubling time and later in vitro senescence than BMSCs.^12^

### ADSC isolation and preparation

Adipose-derived stem cells are typically isolated from lipoaspirates obtained at liposuction procedures, of which, approximately 400 000 are conducted in the United States annually. Each procedure yields approximately 100 mL to 3 L of lipoaspirate, in which 90% to 100% of ADSCs are viable, which is usually discarded following routine liposuction.^40^ To isolate ADSCs, adipose tissue is digested with collagenase, filtered, and centrifuged. The resulting cell pellet is the SVF, containing stromal cells, including ADSCs, which do not contain the lipid droplet in mature adipocytes and have a fibroblast-like morphology.^38^ Other cell types present include endothelial cells, smooth muscle cells, pericytes, fibroblasts, and circulating cells such as leucocytes, haematopoietic stem cells, and endothelial progenitor cells. White adipose tissue (WAT) depots vary in stem cell content and properties depending on anatomical site. Adipose-derived stem cells of visceral origin have a higher self-renewal capacity^59^ and ADSCs from abdominal superficial regions are more resistant to apoptosis than those from the arm, thigh, or trochanteric depots.^10^ This is hypothesised to be secondary to different levels of apoptotic regulators within cells from different depots, such as the Bcl-2 family, in addition to variations in production of paracrine/autocrine factors, eg, insulinlike growth factor 1 (IGF-1).^60^ A recent study demonstrated that superficial abdominal cells have higher G3PD activity, aP2, peroxisome proliferator–activated receptor γ (PPAR-γ), and C/EBP-α expression compared with other depots, which may contribute to their resistance to apoptosis.^61^ However, the greatest numbers of stem cells are isolated from the arm when compared with depots such as the thigh, abdomen, or breast, postulated to be secondary to this depot having the highest PPAR-γ2 expression.^61,62^ The optimum WAT depot ADSC harvest and recovery has yet to be elucidated.^63^

### ADSC characteristics

The immunophenotype of ADSCs is >90% identical to that of BMSCs.^14^ One significant difference between the cell types is the presence of the glycoprotein CD34 on the ADSC cell surface.^63–65^ Adipose-derived stem cells show uniformly positive expression for stem cell markers CD34, CD44, CD73, CD90, and CD105^12^ and are negative for CD19, CD14, and CD45. They are positive for pericytic markers CD140a and CD14b and the smooth muscle marker α-smooth muscle actin. Adipose-derived stem cells secrete growth factors such as vascular endothelial growth factor (VEGF), hepatocyte growth factor, fibroblast growth factor 2 (FGF-2), and IGF-1, all of which are involved in angiogenesis and adipose tissue regeneration.^54,64^ As ADSCs exhibit a similar cell surface immunophenotype as pericytes, it is thought that ADSCs reside within the perivascular region of adipose tissue, between mature adipocytes and adipose extracellular matrix (ECM) near small capillaries.^14,66^

The transition of a multipotent ADSC into a mature adipocyte occurs in 2 stages. First, by determination and differentiation of the stem cell into a preadipocyte, with subsequent terminal differentiation into a mature adipocyte characterised by accumulation of a single lipid droplet within the cell.^54^ This is regulated by the nuclear transcription factor PPAR-γ. The transcriptional programme activated by PPAR-γ is responsible for the regulation of expression of hormone-sensitive lipase, adiponectin, and fatty acid–binding protein 4 (FABP-4).^11^ Insulinlike growth factor 1 stimulates the first stage of adipogenesis. Glucocorticoids, insulin, and growth hormone play a role in the stimulation of the early and late phases of adipogenesis.^40^ Mature adipocytes are terminally differentiated cells with limited capacity for self-renewal and replacement of mature adipocytes.^63^ The responsibility for tissue homeostasis and cell renewal as a result of cells lost due to maturation, damage, or ageing in mature adipose tissue lies with ADSCs.^67^ As ADSCs originate from the SVF of digested adipose tissue, they also have the ability to differentiate into vascular endothelial cells and also produce the pro-angiogenic growth factor VEGF, which would be advantageous in the process of vascularising an engineered tissue construct.^10^

Due to these characteristics, ADSCs hold considerable potential for the regeneration of fat tissue in reconstructive surgery and can be used as both autologous and allogenic grafts in this context.

## ADSCs and Breast Surgery

### Fat grafting

Autologous fat grafting has been successfully used in the clinical setting for breast augmentation, filling small-volume defects post–breast-conserving therapy^68–72^ and contour defects in implant-based breast reconstructions.^73,74^ Although promising aesthetic outcomes have been demonstrated in this setting, the larger volume of adipose tissue required to reconstruct the breast mound post-mastectomy is more challenging.^75^ Autologous fat grafting has had limited success in breast reconstruction with resorption rates ranging from 25% to 80% and complications such as fat necrosis, oil cyst formation, and microcalcifications in patients receiving autologous fat transfer in addition to a primary reconstructive procedure, eg, LD flap^76^ or as a filler for small-volume defects post-BCS.^77–79^ In an attempt to reduce the rate of resorption, cell-assisted lipotransfer, first described by Matsumoto et al^80^ in 2006, involves enrichment of autologous lipoaspirates with ADSCs prior to grafting. Enrichment of autologous fat lipoaspirates with ADSCs which have been expanded ex vivo has had more successful outcomes in terms of volume retention, likely as a result of superior graft maintenance due to increased vascularisation and collagen synthesis within the graft.^14^ Kolle et al demonstrated residual fat volume of >80% in 10 patients over 121 days using abdominal lipoaspirate enriched with ADSCs that had been expanded ex vivo for 14 days prior to reimplantation into the upper posterior arm. Compared with controls, there were higher amounts of adipose tissue, less necrotic tissue, and newly formed connective tissue in grafts enriched with ADSCs.^81^ Yoshimura et al conducted a study in 40 healthy patients undergoing cosmetic breast augmentation, where a mean volume of 270 mL of ADSC-enriched fat was injected into the breast. They reported minimal post-operative atrophy of the injected fat which did not change significantly more than 2 months. Small cystic formations and microcalcifications were observed in some cases; however, the microcalcifications were readily distinguished as benign radiologically. Post-operative computed tomographic and magnetic resonance imaging images showed that transplanted fat tissue survived and breast volume stabilised 2 to 3 months post-operatively. These data indicate that cell-assisted lipotransfer is effective for small-volume breast defects.^82^

### Tissue-engineered constructs

Recreating the breast mound post-mastectomy is likely to require long-term maintenance of larger tissue volumes in engineered grafts supported by a biocompatible scaffold.^28^ There has been limited success with ‘scaffold free’ techniques. This approach involves inducing ADSCs to differentiate into adipocytes and supplementation of culture media with ascorbic acid, to stimulate the production and organisation of ECM to form manipulatable sheets which can be assembled into thicker adipose constructs. Such constructs produced a thickness of 140 ± 14 µm after superimposing 3 adipose sheets.^83^ For scaffold-based tissue-engineered constructs, correct scaffold material and design selection will be paramount in overcoming the obstacles of volume retention and vascularisation. Current tissue engineering strategies involve 2-dimensional or 3-dimensional (3D) natural or synthetic scaffold biomaterials that may or may not be seeded with MSCs.^54^

Scaffolds allow for the culture of cells in a 3D microenvironment, more accurately mimicking native tissue in vivo. The ‘ideal’ scaffold is one that allows for the production of ‘native-like tissue’, with similar physical and biochemical properties of the tissue it is replacing. Choice of scaffold material is a key consideration in the regeneration of specific tissue types (Table 2). Biomaterials act as the biochemical and biophysical environment to tune the cell response for the specific tissue engineering requirement. The properties of biomaterials (eg, mechanical and chemical functionality) affect phenomena such as cell adhesion, proliferation, and differentiation.^54^ The ideal scaffold is also biocompatible, preventing the occurrence of a long-term immune reaction. A highly porous structure is required for vascular ingrowth and cell differentiation. Adipose-derived stem cells undergo morphologic alterations during differentiation to mature adipocytes which includes an increase in diameter from approximately 10 to 100 µm.^84^ Pores within the scaffold must be of adequate size to accommodate changes such as these. A scaffold’s stiffness is an important consideration, in that it must be capable of maintaining its structural integrity despite handling during surgical insertion and physiological forces in vivo, yet flexible so that ingrowth of new tissue and vascular structures is possible. In addition to this, biomaterial stiffness influences ADSC differentiation, eg, when stem cells are encapsulated in polycaprolactone (PCL), they are more likely to differentiate towards bone, tendon, and cartilage over other tissue types.^85^ Biomechanical properties of the biomaterial must be adjustable to regulate the cellular microenvironment. Degradation properties of a biomaterial are imperative; an ideal scaffold should remain intact for sufficient time for new tissue to form but degrade at a sufficient rate that new ECM can be formed and tissue regenerated.^86^ Generally, scaffolds are composed of biomaterials in the form of sponges, hydrogels, 3D or bioprinted constructs, and electrospun scaffolds (Table 2).

Biomaterials can be naturally or synthetically derived. Natural biomaterials used in adipose tissue engineering include collagen, silk, alginate, and gelatin (Table 1). The principle advantage of these biomaterials is their biocompatibility. There are significant differences in the biochemical properties of these biomaterials, eg, collagen, a major component of in vivo microenvironments, is capable of interaction with ADSCs via integrins, unlike alginate, as it does not exist in native ECM and thus does not interact with stem cells.^85^ Von Heimburg et al investigated freeze-dried collagen sponges seeded with preadipocytes. These were implanted into immunodeficient mice and preadipocytes differentiated to mature adipocytes in vivo. The constructs were explanted at 3 and 8 weeks and histologic analysis revealed adipose tissue with rich vascularisation attached to the scaffold beneath a thin capsule layer of fibrovascular tissue. This study highlighted the need for the correct scaffold pore size as scaffolds with smaller pore sizes were unable to support preadipocytes differentiation to mature adipocytes. Developing adipocytes have the potential to grow to a diameter of 100 µm. A narrow pore size can restrict growth and differentiation of preadipocytes.^87^ A study on HYAFF11 sponges, a derivative of hyaluronic acid, concluded that these were superior to collagen sponges regarding cellularity achieved in adipose tissue engineering.^88^ This has been identified as a suitable scaffold material for the culture and in vivo differentiation of ADSCs.^89,90^

**Table 1.** Natural biomaterials previously studied as scaffolds in adipose tissue engineering.

Scaffold	Scaffold	Advantages/disadvantages	Clinical data	References
Collagen	Sponge, injectable microbeads, hydrogel	Advantages ADSC differentiation and vascularisation in vivo Can be modified by addition of growth factors Porosity can be modified Suturable Disadvantages Capsule formation evident in vivo Rapid degradation Low mechanical strength	Collagen sponge impregnated with bFGF for the treatment of chronic skin ulcers	38,54,87,91-93
Hyaluronic acid derivatives	Sponge, hydrogel	Advantages Higher cell density with more homogeneous spread than collagen sponge Well-differentiated adipocytes and large amounts of ECM Disadvantages More expensive than collagen	ADSCs seeded onto cellular biohybrid ADIPOGRAFT and implanted subcutaneously	88-90,94,95
Silk	Disc, hydrogels, sponge, thin films, tubes	Advantages Good mechanical strength Low immunogenicity Silk protein bioengineering allows for expression of growth, differentiation, and angiogenic factors Retain size and porous structure long term due to slow Proteolytic degradation Does not require cross-linking Disadvantages Stability of degradation products unknown	Silk used as a surgical scaffold in soft tissue reconstruction, eg, 2-stage implant breast reconstruction and in the repair of the abdominal wall	54,96-101
Gelatin	Coating, hydrogel, bioprinting, sponge	Advantages Non-toxic Enables delivery of growth factors Disadvantages Rapid degradation Weak mechanical strength Often requires combination with another scaffold biomaterial	Used in conjunction with collagen sponge for treatment of chronic skin ulcers	54,93,102-105
Alginate	Hydrogel, microsphere, bioprinting	Advantages Incorporates well with surrounding tissue Addition of growth factors possible Can be used in combination with other scaffolds Disadvantages Rapid degradation Weak mechanical strength	Alginate hydrogels used as a vehicle for stem cell delivery in the treatment of myocardial infarction	54,106-109

Abbreviations: ADSC, adipose-derived stem cell; bFGF, basic fibroblast growth factor; ECM, extracellular matrix.

Synthetic scaffolds studied in this field include polyglycolic acid (PLGA), polyethylene glycol, PCL, and poly-l-lactic acid (Table 2). Properties such as mechanical strength and stiffness are easily modifiable in synthetic scaffolds. Addition of growth factors and ECM components is also readily possible making these biomaterials very flexible for use in tissue engineering. Patrick et al was one of the first groups to investigate the use of scaffolds in adipose tissue regeneration. They reported the isolation and culture of preadipocytes on a PLGA scaffold which was then successfully implanted into a murine model. Despite initial success, with good adipose tissue formation evident in vivo at 2 months, a decrease in adipose tissue was seen at 3 months with complete disappearance of all adipose tissue and PLGA scaffold at 12 months.^110–112^ More recently, 3D-printed patient-specific breast scaffolds with a poly-lactide polymer have been investigated. These were scaled down to be implanted in mice, seeded with human umbilical cord perivascular cells, and cultured for 6 weeks. The resulting constructs were seeded with human umbilical vein endothelial cells (HUVECs) and subcutaneously implanted in athymic nude mice for 24 weeks. Explanted samples were well-vascularised constructs of adipose tissue without necrosis, inflammation, or cysts. There was an increase in adipose tissue produced from 37.17% to 62.3% between weeks 5 and 15. This further increased to 81.2% between weeks 15 and 24.^113^

**Table 2.** Synthetic biomaterials previously studied as scaffolds in adipose tissue engineering.

Poly(l-lactide-co-glycolide) (PLGA)	Synthetic	3D printing, sponge, injectable spheres, hydrogel	Advantages Biodegradable Modifiable by altering biomechanical and biochemical properties Disadvantages Degradation products cause inflammation Scaffold surface requires modification for ADSC growth and differentiation to occur Capsule formation in vivo Short degradation time	54,111,112,114-118
Polycaprolactone (PCL)	Synthetic	Electrospun mesh, 3D printing, sponge	Advantages Modifiable by altering biomechanical and biochemical properties Good mechanical strength Angiogenesis in ADSC-seeded and ADSC-unseeded constructs Disadvantages Unpredictable degradation Mammalian cell attachment is limited due to its hydrophobicity	54,102,119-122
Polyurethane	Synthetic	Sponge	Advantages Adipogenesis and angiogenesis evident in vivo Elastic Disadvantages Capsule formation evident in vivo	119,123,124
Polypropylene	Synthetic	Mesh	Advantages Good biocompatibility No inflammatory reaction Maintains good dimensional stability after implantation Easily tailored to desired shape Disadvantages Unabsorbable	125
Polylactic acid	Synthetic	Sponge, fleece	Advantages Good mechanical strength Surface modification possible Disadvantages Rapid degradation	54,102,113
Polyethylene glycol (PEG)	Synthetic	Hydrogel	Advantages Low toxicity Water soluble and degradable Promotes adipose tissue regeneration Disadvantages Weak mechanical strength Rapid degradation Requires cross-linking	54,126

Abbreviations: 3D, 3-dimensional; ADSC, adipose-derived stem cell.

More recently, biological scaffolds such as decellularised ECM (dECM) have been studied (Table 3). They generate a minimal immunologic and inflammatory response and provide an accurate mimicry of the native tissue microenvironment by preserving the structure of organised tissue and acting as a natural template for the remodelling of regenerated tissue. Scaffolds exist in different biomaterials and different formats based on the individual requirements of the tissue to be regenerated. Pati et al^127^ successfully bioprinted a 3D cell laden construct with dECM that showed high cell viability and functionality. A similar biomaterial adipose tissue construct was implanted into a mouse model, which demonstrated positive tissue infiltration, constructive tissue remodelling, and adipose tissue formation. Decellularised adipose tissue (DAT) also holds promise as an adipogenic bioscaffold. One study seeded ADSCs onto DAT bioscaffolds and implanted them into female Wistar rats. At explantation at 12 weeks, 56.1% ± 9.2% of the ADSC-seeded DAT had been remodelled into mature adipose tissue. There was a higher density of blood vessels within the areas of the implant that had been remodelled into mature adipose tissue.^128^

**Table 3.** Biological scaffolds previously studies as scaffolds in adipose tissue engineering.

Adipose-decellularised ECM	Natural/biological	Bioprinted, injectable microparticles, hydrogel, 3D printing	Advantages Xenogenic implantation does not cause inflammatory reaction ECM provides the microenvironment for cells to respond to cues for cellular function and activity Well maintained 3D architecture and biochemical composition after decellularisation Adipogenesis and angiogenesis in vivo Can be used in combination with other scaffold biomaterials Disadvantages Not mass-producible Decellularisation technique is complicated and time-consuming	127–132

Abbreviations: 3D, 3-dimensional; ECM, extracellular matrix.

Vascularisation of regenerated tissue is one of the primary challenges of tissue engineering. Several methods of providing these constructs with adequate blood supply have been investigated. The scaffold within regenerated tissue can play a prominent role in this regard, as scaffold stiffness and porosity are known to influence angiogenesis.^133^ In addition, ADSCs themselves are implicated in the regulation of neovascularisation through their modulation of the ECM, or scaffold, by matrix metalloproteinases (MMPs).^134^ Some studies have sought to aid vascularisation by the addition of HUVECs to ADSC and scaffold constructs.^135,136^ Chhaya et al seeded HUVECs onto their adipose tissue construct prior to implantation into a murine model. They observed a 62.3% increase in adipose tissue with the formation of a functional capillary network within the tissue.^113^ Vascular pedicles have been used as additional support for an engineered adipose construct within a chamber to facilitate large-scale adipose tissue engineering.^137^ In this setting, a chamber allows the vascular pedicle to induce intense vasculogenesis to maximise cell survival.^84^ Dolderer et al^138^ demonstrated a 10% to 15% increase in adipose tissue volume over a 20-week period using this approach; there was no evidence of hypertrophy, fat necrosis, or atypical changes in the regenerated tissue. Findlay et al^114^ was successful in generating up to 56.5 mL of adipose tissue by implanting bilateral 78.5-mL chambers subcutaneously in the groin of a pig which enclosed a fat flap based on the superficial circumflex iliac pedicle for 22 weeks. Implantation of a scaffold prior to ADSC/adipose tissue to allow for ingrowth of new vessels among the scaffold has been investigated. ‘Additive biomanufacturing’ used delayed fat injection into a custom-made scaffold implanted in minipigs for 24 weeks after a period of prevascularisation. The prevascularisation + lipoaspirate group had the highest relative area of adipose tissue on explantation (47.32% ± 4.12%) which was similar to native breast tissue (44.97%±14.12%).^122^ These studies represent the largest volumes of adipose tissue engineering in vivo.

The transition to large animal studies and the generation of larger, more clinically relevant volumes of adipose tissue present an exciting prospect for translation of a tissue-engineered breast reconstruction to the clinical setting. However, the evidence for the oncological safety of using ADSCs in patients who have been treated for breast cancer is limited and requires further investigation before the knowledge and techniques generated through these studies can be considered for application in post-mastectomy reconstruction.^39^

## Oncologic Safety Considerations

Despite the promising early results of ADSCs in breast lipofilling and small-volume reconstruction, its application towards post-oncologic reconstruction should be approached with caution. The concern regarding use of autologous ADSCs in this setting stems from the very same characteristics which make them so attractive for tissue regeneration. Adipose-derived stem cells may potentially contribute to stromal support for cancer cells and deliver locally inflammatory cytokines and/or growth factors, thus facilitating residual cancer cell survival and growth.

### Tumour microenvironment

Breast cancer grows in close anatomical proximity to adipose tissue. The ‘tumour microenvironment’ consists of a complex signalling network which influences the behaviour of both resident stem cells and tumour cells.^46^ It is composed of all cells surrounding the tumour including endothelial, inflammatory and immune cells, adipocytes, myoepithelial cells, and fibroblasts in conjunction with their ECM.^139,140^ Understanding the complexity of tumour-stromal interactions will enhance our understanding of how ADSCs may interact with the tumour microenvironment. Numerous autocrine, paracrine, and exocrine pathways in this environment have been described as a role-playing factor in breast cancer.^47^

A subset of adipocytes known as ‘cancer-associated adipocytes’ (CAAs) have been shown to play an active role in tumour progression and metastasis.^141^ Cancer-associated adipocytes are mature adipocytes that have dedifferentiated into preadipocytes through loss of their lipid droplet and adopted a fibroblastic morphology.^11^ This cell type increases tumour growth, tumour invasion via greater epithelial-mesenchymal transition (EMT)^14^ and results in a radio-resistant breast cancer phenotype.^13^ Several inflammatory cytokines are thought to be involved in this process, eg, tumour necrosis factor α (TNF-α), interleukin (IL)-1, IL-6, IL-11, leukaemia inhibitory factor, IFN-γ, oncostatin M, and ciliary neurotrophic factor. They have been observed to both inhibit stem cell commitment and differentiation towards adipogenesis and may be implicated in adipocyte dedifferentiation.^67^

In order for cancer to progress, it requires stem cells and partly differentiated progenitor cells to be recruited from local and distant sites and angiogenesis, both of which occur due to release of factors by the inflammatory and hypoxic tumour microenvironment.^142^ Adipose-derived stem cells have a role in angiogenesis and localise to sites of injury and contribute to revascularisation.^64^ Epithelial-mesenchymal transition plays a major role in tumour development and benign resident and stromal cells recruited to the area are implicated in early cancer development and metastasis.^39^ These stromal cells within the tumour microenvironment are collectively known as ‘cancer-associated fibroblasts’. They secrete pro-angiogenic and anti-apoptotic factors, contributing to tumour development. Zhang et al^59^ demonstrated that ADSCs mobilise and migrate through the systemic circulation to distant tumours resulting in acceleration of tumour growth. This action appears to be unique to ADSCs and is not observed in similar models using bone marrow-derived or lung-derived MSCs.^143^ It remains unclear whether ADSCs used in wound repair are capable of migration to distant tumours. Altman et al investigated whether ADSCs had any effect on the growth and progression of distant tumours when applied to a skin wound; comparing tumour growth in vivo (murine model) when breast cancer cells and ADSCs were co-injected and when the ADSCs were introduced on an ADM at a distant skin wound. Although there was an increase in tumour volume when ADSCs were co-injected, there was no such effect observed in cases where the ADSCs were introduced at the skin wound, indicating that that the wound microenvironment is capable of retaining ADSCs and preventing their migration to distant malignant sites.^144^

Adipose-derived stem cells, both local and those recruited to a breast tumour site, are capable of dedifferentiation into CAAs. Several genes involved in cell growth, ECM deposition/remodelling, and angiogenesis are expressed at higher levels in local breast ADSCs than those isolated from adipose tissue or bone marrow, suggesting that the breast adipose depot plays a more intimate role in breast cancer progression.^145^ Coculture of breast cancer cells and preadipocytes prevents adipogenic differentiation, supporting the hypothesis that ADSCs are part of the CAA population within breast cancer tumours.^146^

Extracellular matrix secreted by adipocytes also has a role in breast cancer progression.^147–150^ Adipose tissue ECM is rich in collagen VI,^151^ which is upregulated in tumorigenesis and promotes GSK3β phosphorylation and increased β-catenin activity in breast cancer cells. Breast tumour growth has been shown to be reduced in a collagen VI–deficient murine model. Breast cancer cells cocultured with adipocytes and injected subcutaneously in the mammary fat pad of a nude mouse resulted in larger tumours than breast cancer cells cocultured with fibroblasts and Matrigel.^152^ Matrix metalloproteinases are enzymes involved in the degradation of ECM proteins during growth and tissue turnover. Higher levels of MMP-11 are expressed by adipocytes at the invasive front of human breast cancers secondary to ECM remodelling in this area. MMP-11 is a negative regulator of adipogenesis and may be responsible in part for the ‘dedifferentiation’ of adipocytes.^13^ Certain cell surface markers, eg, CD44, mediate reorganisation of ECM components, by anchoring MMPs to the cell surface. Therefore, ADSCs play a direct role in ECM remodelling occurring during tumour growth.^12^ Adipose-derived stem cells are involved in the desmoplastic reaction occurring within tumours through their involvement with MMPs. Desmoplasia results in the recruitment of myofibroblasts, a cell type frequently detected in breast cancer tumour stroma. Adipose-derived stem cells express α-smooth muscle actin, a marker for myofibroblasts, suggesting that ADSCs are a source of tumour myofibroblasts.^14^ These intricate interactions within the tumour microenvironment illustrate how ADSCs may create a favourable milieu for tumour growth.

### ADSC secretome

**Figure 1.**
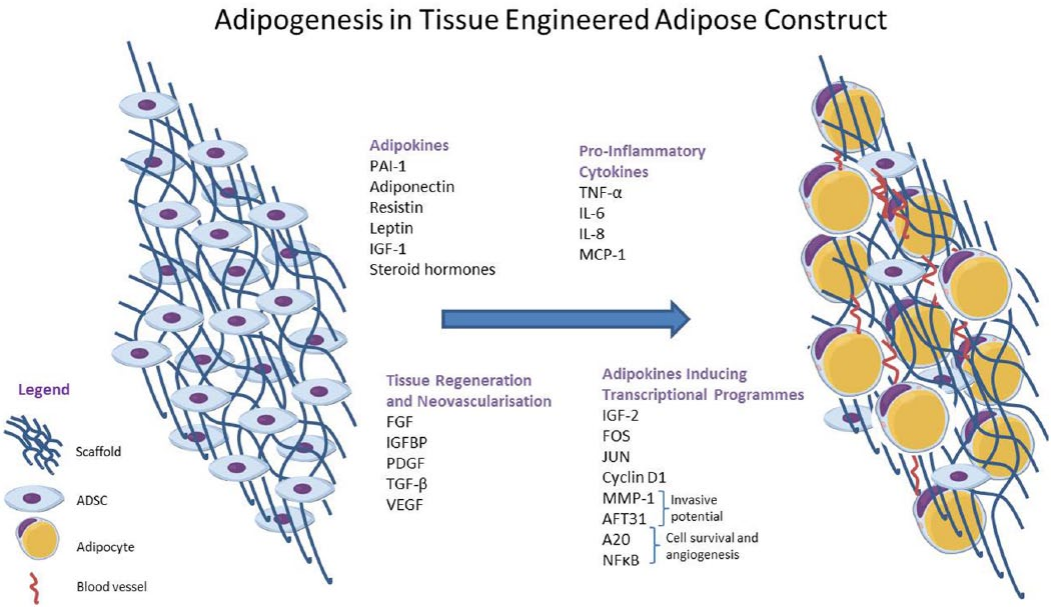
Adipogenesis in tissue-engineered adipose construct and produced adipokines. ADSC indicates adipose-derived stem cell; FGF, fibroblast growth factor; IGF, insulinlike growth factor; IGFBP, IGF-binding protein; IL, interleukin; MMP-1, matrix metalloproteinase 1; NF-κB, nuclear factor κB; PDGF, platelet-derived growth factor; TGF-β, transforming growth factor β; TNF-α, tumour necrosis factor α; VEGF, vascular endothelial growth factor.

Adipose-derived stem cells possess tumour-supporting functions through provision of migratory cells which secrete trophic factors, increasing vascularisation and contributing to survival and proliferation of malignant cells.^140^ Adipose tissue secretes cytokines known as adipokines, eg, TNF-α, IL-6, IL-8, PAI-1, MCP-1, adiponectin, resistin, leptin, insulin growth factor, and steroid hormones, some of which have been studied in relation to cancer,^142^ eg, leptin upregulates activity in signalling pathways in breast cancer tumours that play a role in proliferation (Figure 1).^13^

Many adipokines are pro-inflammatory, are secreted in increasing amounts in obese individuals, and are involved in the promotion of tumour growth, locally at the tumour site and, via communication with distant sites, in particular TNF-α, IL-6, IL-8, and MCP-1.^11,13,59^ Factors known to play a significant role in tissue regeneration, neovascularisation, carcinogenesis, and tumour progression found in high-risk patients, expressed by MSCs and ADSCs, include FGF, ILs, IGF-binding protein, platelet-derived growth factor, transforming growth factor β (TGF-β), TNF-α, and VEGF.^39^

Adipokines induce transcriptional programmes implicated in promoting tumorigenesis which include increased cell proliferation through IGF-2, FOS, JUN, and cyclin D1; invasive potential through MMP-1 and AFT3l; cell survival via A20 and nuclear factor κB; and angiogenesis.^152^

Adipose-derived stem cells may also influence tumour growth and progression through exertion of immunomodulatory effects on T cells within the tumour microenvironment due to the secretion of cytokines such as prostaglandin E_2_, TGF-β1, indoleamine 2,3-dioxygenase, hepatocyte growth factor (HGF), and inducible nitric oxide synthase. Adipose-derived stem cells may be responsible for abnormal CD4^+^ T-cell activation and function.^15^ Razmkhah et al investigated the expression of IL-4, IL-10, and TGF-β1 in ADSCs isolated from breast tissue in patients with cancer and healthy controls and whether these cytokines had an influence on peripheral blood lymphocytes. Messenger RNA expression of IL-10 and TGF-β1 in ADSCs from patients with cancer was higher than those isolated from healthy patients. The conditioned media from ADSCs of patients with stage III disease was used to culture ADSCs from healthy patients and caused IL-4 and IL-10 expression to increase. Therefore, ADSCs may assist in protecting the breast cancer from anti-tumour immune responses by providing a source of anti-inflammatory cytokines within the tumour microenvironment and their potential to act as regulatory T cells.^15^

Breast adipose tissue functions in oestrogen biosynthesis and high local levels of oestrogen are related to breast cancer development and progression.^153^ Oestrogen upregulates growth factors such as epidermal growth factor receptor and Akt phosphorylation, sustaining breast cancer growth.^154^ Oestrogen plays a role in the increased aggressiveness of breast cancer in obese individuals.^155^ Adipose-derived stem cells isolated from abdominal adipose tissue of those with a body mass index >30 enhance breast cancer cell line proliferation and tumorigenicity in vitro and in vivo. Changes in the oestrogen receptor alpha and progesterone receptor gene expression profile correlated with these changes. Obesity caused changes in several adipogenic genes including leptin, and women with ER+/PR+ tumours that had high leptin expression had a poorer prognosis.^156^

### The effect of ADSCs on breast cancer: in vitro and in vivo evidence

As discussed above, ADSCs have the potential to influence the behaviour of breast cancer cells due to secreted adipokines and their effect on the tumour microenvironment. However, there are conflicting reports on the manner in which this influence is exerted.

The role of adipokines was demonstrated by Dirat et al^141^ who reported increased invasiveness of both human and murine breast cancer cells associated with overexpression of proteases, including MMP-11, and pro-inflammatory cytokines (IL-6, IL-1β), when cocultured with adipocytes. Zhang et al^157^ demonstrated that ADSCs increased the motility of MCF-7 breast cancer cells in vitro through the secretion of the chemokine CCL5.

However, it has been suggested that ADSCs may only promote the growth and progression of active breast cancer cells and do not activate dormant residual breast cancer cells; therefore, the use of ADSC regenerative therapies should therefore be delayed until there is no evidence of active disease.^158^

Indeed, there is a lack of consensus on the reported effects of ADSCs on tumour behaviour as some studies have demonstrated an ability by ADSCs to *inhibit* tumour growth in vitro. Adipose-derived stem cells are capable of inducing cell death in pancreatic adenocarcinoma, hepatocarcinoma, colon, and prostate cancers.^159,160^ Adipose-derived stem cells cultured at high density and their conditioned media have been shown to be capable of suppressing the growth of MCF-7 cells in vitro, as a result of IFN-β expression by ADSCs in high-density culture.^161^

There is a similar discordance in results from in vivo studies. Increased tumour growth and metastasis in a murine model was observed when ADSCs from WAT were co-injected with triple-negative human breast cancer cells. Tumour progression was similar in the groups that were co-injected with human breast cancer cells and unprocessed lipoaspirate and those co-injected with human breast cancer cells and purified CD34^+^ WAT ADSCs, suggesting that most of the tumour progression effects of human WAT are due to the ADSC fraction. A follow-up metastasis study demonstrated that mice which had a primary breast cancer tumour removed and were injected with ADSCs alone had a higher rate of axillary and lung metastasis than mice which had CD34− cells injected post-resection. Immunohistochemistry revealed that human cells generated from ADSCs were incorporated into the tumour vasculature. These effects have never been observed using bone marrow–derived stem cells, suggesting that these functions are unique to ADSCs.^53^

Conversely, the ability of ADSCs to inhibit MDA-MB-231 breast adenocarcinoma cells was demonstrated by Sun et al.^162^ The authors using a murine cancer model similar to prior studies demonstrated that ADSCs did not appear to increase tumour progression or metastasis and actually had the effect of inhibiting breast cancer cells by apoptosis.

Experimental data, both in vitro and in vivo, are conflicting regarding the effect of ADSCs on breast cancer, and there is a lack of consensus on this subject. Data from their use in the clinical setting must also be considered when evaluating the oncological safety of their use in patients with breast cancer.

## ADSCs – Clinical Use in Patients With Breast Cancer

As outlined above, the properties of ADSCs which are advantageous tissue regeneration, including immune-modulatory, pro-survival, pro-angiogenic, and anti-apoptotic effects, immunosuppression, tissue growth, and cellular homing are also dysregulated in tumour progression and metastasis, thus raising questions regarding the oncologic safety of these cells in breast reconstruction post-mastectomy.^39,163,164^ In addition to experimental mechanistic data, the clinical evidence relating to the safety of ADSC use in patients with breast cancer has been assessed in both retrospective and prospective series of patients undergoing ADSC-enhanced fat grafting (Table 4).

**Table 4.** Studies of breast cancer recurrence post-autologous fat grafting procedures.

Author	Type of study	Year of publication	No. of patients	Length of follow-up, mo	Oncologic procedure or cosmetic	Rate of recurrence, %	Reference
Delay et al	Retrospective cohort study	2009	880	120	Both	1.5/y	77
Rigotti et al	Retrospective cohort study	2010	137	36	Oncology	3.6	171
Rietjens et al	Prospective cohort study	2011	158	6	Oncology	0	172
Petit et al	Multicentre retrospective cohort study	2011	513	19.2	Oncology	5.6	173
Sarfati et al	Cohort study	2011	28	17	Oncology	0	174
Petit et al	Prospective matched cohort study	2012	321	26	Oncology	2.5	165
Pérez-Cano et al	Single-arm prospective multicentre clinical trial	2012	71	12	Oncology	0	175
Petit et al	Matched cohort study	2013	59	38	Intra-epithelial neoplasia	18	166
Riggio et al	Observational study	2013	60	120	Oncology	7.25	176
Ihrai et al	Retrospective cohort study	2013	64	46	Oncology	3.1	177
Brenelli et al	Prospective cohort study	2014	59	34.4	Oncology – all breast-conserving surgery	4	178
Semprini et al	Cohort study	2014	151	45	Oncology	0	179
Gale et al	Case-control study	2015	328	32	Oncology	Local: 0.95 Regional: 0.95 Distant: 3.32	180
Masia et al	Retrospective case-control study	2015	100	29	Oncology	2.8	181
Pinell-White et al	Matched cohort study	2015	51	50.4	Oncology	5.9	182
Silva-Vergara et al	Prospective cohort study	2016	195	31	Oncology	3.1	183
Kronowitz et al	Matched controlled study	2016	719	60	Oncology	1.3	167
Mestak et al	Prospective cohort study	2016	32	Not reported	Oncology	6.25	170
Kaoutzanis et al	Retrospective cohort study	2016	108	20.2	Oncology	0	184
Garcia et al	Prospective cohort study	2016	37	12	Oncology	0	185

The oncological safety of autologous fat grafting has been assessed in multiple prospective and retrospective series of patients who had undergone BCS or mastectomy (Table 4). Petit et al reported local recurrence rates of 1.35% for the mastectomy group and 2.19% in the BCS group. Only patients with intraepithelial neoplasia (n = 37) who underwent autologous fat grafting demonstrated an increased rate of local recurrence (10.8%).^165^ A follow-up–matched cohort study investigating fat grafting in 59 patients with intraepithelial neoplasia concluded that there is a higher risk of local recurrence in this patient cohort compared with age-matched and stage-matched controls (n = 118).^166^ Although the results of these studies are significant, they are retrospective series in a single centre with small numbers of patients. Several other explanations may exist for this increased rate of recurrence: there was a higher rate of recurrence in those patients with close or positive surgical margins in the study group, and there was an increased rate of recurrence in those with a higher grade tumour. There is no other published study that reports such an increased rate of breast cancer recurrence after autologous fat grafting. Further prospective investigation of the risk that intraepithelial neoplasia poses in autologous fat grafting is required, in larger numbers of patients with longer follow-up. The largest retrospective study to date which focused on ADSCs use in patients with a history of breast cancer was conducted by Kronowitz et al. The authors conducted a retrospective matched controlled study of 719 patients undergoing lipofilling of the breast post-tumour resection. There was no increase in locoregional or systemic recurrence or of a second breast cancer.^167,168^ In a separate retrospective study conducted by Petit et al,^169^ which focused on local recurrence consisted of 370 patients who underwent mastectomy (1.35%) and 143 patients who underwent BCS (2.19%), the authors concluded that there was no difference in recurrence rate of either group when compared with controls. Several other studies showed similar rates of recurrence in patients who solely underwent BCS when compared with controls.^170^ Therefore, there does not appear to be any difference in the rate of recurrence in patients undergoing autologous fat grafting post-BCS or mastectomy and reconstruction.

The RESTORE-2 trial prospectively assessed the oncological safety of ADSC-enriched fat grafting in patients undergoing BCS with defects of up to 150 mL. In total, 67 patients reported high levels of satisfaction with the cosmetic outcomes. No incidences of local recurrence were reported within 12 months of the procedure; however, this is not a sufficient follow-up time to adequately investigate the oncologic safety of this technique.^121^

Delay et al analysed outcomes in 880 patients in a retrospective review, who underwent fat grafting. This review of the practice of 4 surgeons demonstrated that after 10 years of follow-up, there was no increased risk of cancer recurrence or new cancer development.^77^ They also reported that the radiological appearance of the breasts post-lipofilling was usually of normal breast tissue; however, some images showed fat necrosis, which was easily distinguished from neoplastic lesions. Systematic reviews on the topic conclude that autologous fat grafting appears to be oncologically safe with low rates of complications and satisfactory patient and surgeon satisfaction.^186–189^ However, questions remain with many of these previous mentioned studies as limited information is given about tumour size, lymph node status, and chemotherapy regimens, all of which would affect the locoregional recurrence (LRR) rate. Another key factor is the follow-up time, which varied considerably between studies. The largest study published by Kronowitz et al had a follow-up time of 5 years; in comparison, Petit et al reported a follow-up time of a little more than 2 years.^167,169^ The timing of the autologous fat grafting after surgery is another area for consideration; deciding on an optimal time point post primary surgery for autologous grafting with ADSCs may indeed be the primary decision in preventing recurrence. It is due to these issues that all authors call for well-designed randomised controlled trials with adequate follow-up to adequately address these issues and to exercise caution in performing these procedures in high-risk patients. A phase 3 multicentre randomised controlled trial is currently underway in France with the goal of investigating this issue (GRATSEC NCT01035268).

One further suggested explanation for the discrepancies between basic science and clinical studies in relation to oncological safety is the higher concentration of ADSCs used in vitro than clinically, which raises further concerns for the use of ADSCs in tissue engineering strategies which would require high concentrations of ADSCs to generate large volumes of adipose tissue.^75,186,189–191^

## Adjuvant Therapy Considerations

If ADSC-based breast reconstruction/regeneration approaches are to be translated to the clinical setting for patients with breast cancer, in addition to addressing oncologic safety, the potential effect of tissue regeneration using ADSCs on the efficacy of adjuvant therapy and the effects of adjuvant therapy on the success of tissue regeneration and breast reconstruction also require investigation.

### Cytotoxic chemotherapy

After neoadjuvant chemotherapy for breast cancer, poor wound healing is a significant problem for patients undergoing subsequent tumour resection and reconstruction. It is postulated that chemotherapy influences ADSC’s ability to function effectively. Charon et al were the first to investigate the effect of paclitaxel on ADSCs. They found that paclitaxel inhibits proliferation and differentiation of ADSCs and can induce apoptosis. Paclitaxel can also impair wound healing in chemotherapy patients due to its inhibition of endothelial differentiation in ADSCs.^192^ Harris et al treated ADSCs isolated from human periumbilical fat with paclitaxel at various concentrations in vitro. Adipose-derived stem cells from rats treated with paclitaxel were also investigated. Paclitaxel treatment resulted in increased ADSC apoptosis and decreased cell proliferation and migration and inhibited ADSC multipotent differentiation in both human and rodent cell populations. However, human and rodent ADSCs recovered differentiation abilities after cessation of paclitaxel treatment.^193^ Chen et al^194^ demonstrated that ADSCs induce doxorubicin resistance in MDA-MBA-231 triple-negative breast cancer cells through IL-8 secretion. However, ADSCs are shown to cause increased chemosensitivity to doxorubicin and 5-flourouracil in SKBR3 Her2 breast cancer cell lines.^195^ Beane et al discovered that vincristine, cytarabine, and etoposide all inhibited the proliferative ability of ADSCs, although the authors did note that variability did exist between drug type and concentration. In direct comparison, it was found that ADSCs’ growth or viability was not inhibited by any concentration of methotrexate.^196^

Overall, it would appear that each chemotherapeutic agent interacts with ADSCs in a distinct manner and each would warrant investigation. What is clear, however, is that harvesting and storing ADSCs prior to beginning any chemotherapeutic regime may be the best approach to maximise their benefit in terms of adipose regeneration.

### Targeted therapies

#### Trastuzumab

An in vitro coculture study and in vivo analysis of Her2 breast cancer found that adipocytes play a role in resistance of Her2-overexpressing breast cancer cells to trastuzumab. Whether this effect is exclusive to mature adipocytes or whether ADSCs are capable of similar promotion of breast cancer cell resistance to trastuzumab requires further investigation.^197^

#### Hormonal therapy

Tamoxifen is the most widely used adjuvant hormonal therapeutic agent for the treatment of breast cancer.^198^ Pike et al isolated human ADSCs and treated them with various concentrations of tamoxifen. This resulted in increased apoptosis, decreased proliferation of human ADSCs, and a decrease in differentiation capability into adipocytes and osteocytes and inhibited ability to form cords in Matrigel, suggesting that patients treated with tamoxifen may have decreased ADSC graft survival.^199^ This is a cause for concern and could potentially mean women may not be suitable for ADSC-based tissue regeneration while being treated with tamoxifen. In the clinical setting, Kronowitz et al^167^ showed a significant increased risk of LRR in patients who received hormonal therapy after autologous fat grafting. Although it is unclear what this increased risk is due to, Waked et al^47^ hypothesised that it may be due to a stimulatory effect of hormonal therapy on the communication between breast cancer cells and ADSCs or more worryingly that hormone receptor–positive patients with breast cancer may be at increased risk of LRR post-autologous grafting. This is a hypothesis that also requires further investigation.

#### Radiotherapy

Post-mastectomy radiotherapy can result in complications such as recurrent infection, impaired healing, fibrosis, contracture, and lymphoedema. Adipose-derived stem cells display an element of radio-resistance in comparison with other components of the SVF.^200^ This may be secondary to a superior ability of MSCs to retain their proliferative capacity due to their enhanced repair mechanisms for damaged DNA compared with terminally differentiated cells. Lower metabolic demands of ADSCs compared with mature adipocytes result in protection from hypoxia and apoptosis, preserving them to perform regenerative functions.^200^ There are several possible mechanisms by which injection of ADSCs into a previously irradiated area can overcome radiation-induced injury: ADSC adipogenic differentiation; increasing perfusion of injured tissues through induction and paracrine promotion of angiogenesis; exerting an anti-oxidant effect against hypoxia, ischaemia reperfusion, and reactive oxygen species–induced damage by adipokine release; modulating immune responses, inflammation, and improving wound healing; modulating granulation tissue, fibrosis, ECM remodelling, and improve epithelialisation; secreting lymphangiogenic factors, improving or reversing lymphoedema in damaged tissues; and recruiting endogenous stem cells via a homing chemokine gradient.^200^ A study of cocultured ADSCs and normal human fibroblasts (NHF) exposed to radiation shows promise for the use of ADSCs in breast reconstruction. Monocultures of ADSCs and NHFs showed reduced cell proliferation after radiation exposure; however, reduced impairment of cell proliferation was seen in the cocultured cells after radiation exposure. Gene expression of MMPs was also improved in the cocultured group.^201^ Microvascular endothelial cells were then added to the coculture. The levels of cytokines and adhesion molecules, IL-6, bFGF, ICAM-1, and VCAM-1, in the coculture supernatants were significantly less affected by irradiation than monocultures.^202^

Adipose-derived stem cells improve graft retention in irradiated scalps of mice. Fat grafts supplemented with ADSCs demonstrated superior volume retention, structural qualities, and vascularity.^203^ In a study of the effect of ADSCs on flap survival in irradiated tissues in rats, increased flap viability was observed in the ADSC-injected irradiated group compared with the control radiation only group. The mechanism may be both neovascularisation and vasodilation in addition to endothelial repair.^204^ A clinical study of the treatment of radiotherapy-induced injury by lipoaspirate-containing ADSCs showed improved outcomes for 20 grade 3 or 4 patients on the Late Effects Normal Tissue Task force - Subjective, Objective, Management, Analytic (LENT-SOMA) scale measuring severity of radiation effects, with improvement or remission of symptoms in all 20 patients.^205^

Further investigation of the effects of adjuvant cancer therapies, both cytotoxic and targeted on ADSC-based tissue regeneration, is required before this method of breast reconstruction can be considered for translation into the clinical setting.

## Future Direction

Although there has been significant advancement made in the field of adipose tissue engineering, and in the use of ADSCs as a cell source in this regard, there are several outstanding issues that need to be addressed before adipose tissue engineering can be used to its full potential in breast reconstruction. Most basically, the isolation techniques used to produce lipoaspirate require refinement to optimise cell yield, survival, and viability. Investigation of this has thus far revealed that high-speed centrifugation is harmful to lipoaspirates and that washing the adipose tissue to separate it from blood and infiltration solutions may improve outcomes.^206^ However, as of yet, there is no consensus on the protocol for adipose aspiration.^207^ The optimal adipose depot also needs to be identified. First, as the source of greatest ADSC cell yield, and second, as the one that is oncologically safe. As previously discussed, several genes involved in cell growth, ECM deposition, or remodelling and angiogenesis are expressed at higher levels in local breast ADSCs than those isolated from adipose tissue or bone marrow, suggesting that the breast adipose depot plays a more intimate role in breast cancer progression.

The question of oncological safety of ADSCs needs to be definitively answered through investigation of the adipokines produced by this cell type and elucidating the role that these adipokines play in EMT and the tumour microenvironment. The role of ADSCs in stromal support for tumours will also require scrutiny in future experiments. Patient factors will also influence the oncological safety of ADSCs. Many adipokines are pro-inflammatory and are secreted in increasing amounts in obese individuals and are involved in the promotion of tumour growth, which begs the question, whether ADSCs are used in breast reconstruction, are obese individuals are greater risk of cancer recurrence? In addition, are there any other patient characteristics that place them at a greater recurrence risk?

Timing of reconstruction with ADSCs also requires careful consideration. It has been suggested that ADSCs may only promote the growth and progression of active breast cancer cells and not dormant residual breast cancer cells. Therefore, should the use of ADSC regenerative therapies be delayed until such time that there is no evidence of active disease, and what is the optimal time point post-curative surgery for reconstruction with ADSC technology to prevent recurrence?

Several clinical studies and systematic reviews have concluded that reconstruction techniques using autologous fat and ADSCs are oncologically safe with no increased rate of LRR. However, data such as tumour size, lymph node status, and adjuvant therapy regimens are scant. These factors are highly influential on the rate of recurrence and so need to be studied in greater detail in clinical trials of breast reconstruction techniques involving ADSCs.

Finally, there has been limited investigation of the relationship between ADSCs, adjuvant therapies, such as chemotherapy and radiotherapy, and cosmetic and oncological outcomes. The potential effect of tissue regeneration using ADSCs on the efficacy of adjuvant therapy and the effect of adjuvant therapy on the success of tissue regeneration and breast reconstruction requires elucidation.

## Conclusions

With rates of mastectomy showing no sign of decline, novel, safe, functional, and cost-effective methods of breast reconstruction are required. Adipose tissue has been shown to be a valuable source of MSCs that hold immense potential for modern tissue engineering strategies in the field of breast reconstruction. However, there are still several pertinent research questions outstanding regarding the best adipose tissue depot from which to isolate ADSCs and how to generate and sustain volumes of mature adipose tissue to reconstruct the breast mound. Furthermore, the oncological safety of implanting ADSCs into patients with breast cancer due to the risk of cancer recurrence and what effects do adjuvant therapies have on ADSC isolation and their function on implantation remain to be fully elucidated. Well-designed randomised controlled trials will be required to accurately answer these issues. However, it is currently widely believed that ADSCs will be central to the development of novel future techniques in adipose tissue engineering.

## References

[1] Cancer IARC. GLOBOCAN 2012: Estimated Cancer Incidence, Mortality and Prevalence Worldwide in 2012. Lyon: IARC; 2012.

[2] Albornoz CR Matros E Lee CN et al Bilateral mastectomy versus breast-conserving surgery for early-stage breast cancer: the role of breast reconstruction. Plast Reconstr Surg. 2015;135:1518–1526. 26017588 10.1097/PRS.0000000000001276 PMC4744797

[3] Habermann EB Abbott A Parsons HM Virnig BA Al-Refaie WB Tuttle TM Are mastectomy rates really increasing in the United States? J Clin Oncol. 2010;28:3437–3441. 20548000 10.1200/JCO.2009.27.6774

[4] Dragun AE Huang B Tucker TC Spanos WJ Increasing mastectomy rates among all age groups for early stage breast cancer: a 10-year study of surgical choice. Breast J. 2012;18:318–325. 22607016 10.1111/j.1524-4741.2012.01245.x

[5] Tuttle TM Habermann EB Grund EH Morris TJ Virnig BA Increasing use of contralateral prophylactic mastectomy for breast cancer patients: a trend toward more aggressive surgical treatment. J Clin Oncol. 2007;25:5203–5209. 17954711 10.1200/JCO.2007.12.3141

[6] Mahmood U Hanlon AL Koshy M et al Increasing national mastectomy rates for the treatment of early stage breast cancer. Ann Surg Oncol. 2013;20:1436–1443. 23135312 10.1245/s10434-012-2732-5

[7] Jeevan R Cromwell DA Browne JP et al Findings of a national comparative audit of mastectomy and breast reconstruction surgery in England. J Plast Reconstr Aesthet Surg. 2014;67:1333–1344. 24908545 10.1016/j.bjps.2014.04.022

[8] Kenny P King M Shiell A et al Early stage breast cancer: costs and quality of life one year after treatment by mastectomy or conservative surgery and radiation therapy. Breast. 2000;9:37–44. 14731583 10.1054/brst.1999.0111

[9] Heneghan H Prichard R Lyons R et al Quality of life after immediate breast reconstruction and skin-sparing mastectomy – a comparison with patients undergoing breast conserving surgery. Eur J Surg Oncol. 2011;37:937–943. 21899982 10.1016/j.ejso.2011.08.126

[10] Mizuno H Tobita M Uysal AC Concise review: adipose-derived stem cells as a novel tool for future regenerative medicine. Stem Cells. 2012;30:804–810. 22415904 10.1002/stem.1076

[11] Nieman KM Romero IL Van Houten B Lengyel E Adipose tissue and adipocytes support tumorigenesis and metastasis. Biochim Biophys Acta. 2013;1831:1533–1541. 23500888 10.1016/j.bbalip.2013.02.010 PMC3742583

[12] Hass R Otte A Mesenchymal stem cells as all-round supporters in a normal and neoplastic microenvironment. Cell Commun Signal. 2012;10:26. 22943670 10.1186/1478-811X-10-26 PMC3444900

[13] Wang Y-Y Lehuédé C Laurent V et al Adipose tissue and breast epithelial cells: a dangerous dynamic duo in breast cancer. Cancer Lett. 2012;324:142–151. 22643115 10.1016/j.canlet.2012.05.019

[14] Bielli A Scioli MG Gentile P et al Adult adipose-derived stem cells and breast cancer: a controversial relationship. Springerplus. 2014;3:345. 25089245 10.1186/2193-1801-3-345 PMC4117859

[15] Razmkhah M Jaberipour M Erfani N Habibagahi M Talei AR Ghaderi A Adipose derived stem cells (ASCs) isolated from breast cancer tissue express IL-4, IL-10 and TGF-β1 and upregulate expression of regulatory molecules on T cells: do they protect breast cancer cells from the immune response? Cell Immunol. 2011;266:116–122. 20970781 10.1016/j.cellimm.2010.09.005

[16] Harnett A Smallwood J Titshall V Champion A Diagnosis and treatment of early breast cancer, including locally advanced disease – summary of NICE guidance. BMJ. 2009;338:598–600. 10.1136/bmj.b438 PMC3266859 19244302

[17] Jagsi R Jiang J Momoh AO et al Trends and variation in use of breast reconstruction in patients with breast cancer undergoing mastectomy in the United States. J Clin Oncol. 2014;32:919–926. 24550418 10.1200/JCO.2013.52.2284 PMC4876312

[18] Albornoz CR Bach PB Mehrara BJ et al A paradigm shift in US Breast reconstruction: increasing implant rates. Plast Reconstr Surg. 2013;131:15–23. 23271515 10.1097/PRS.0b013e3182729cde

[19] Farhangkhoee H Matros E Disa J Trends and concepts in post-mastectomy breast reconstruction. J Surg Oncol. 2016;113:891–894. 26876921 10.1002/jso.24201 PMC5540647

[20] Manahan MA Prucz RB Shridharani SM Baltodano PA Rosson GD Long-term follow-up of changing practice patterns in breast reconstruction due to increased use of tissue expanders and perforator flaps. Microsurgery. 2014;34:595–601. 24665002 10.1002/micr.22245

[21] Kwok AC Goodwin IA Ying J Agarwal JP National trends and complication rates after bilateral mastectomy and immediate breast reconstruction from 2005 to 2012. Am J Surg. 2015;210:512–516. 26054659 10.1016/j.amjsurg.2015.03.019

[22] Gamboa-Bobadilla GM Implant breast reconstruction using acellular dermal matrix. Ann Plast Surg. 2006;56:22–25. 16374090 10.1097/01.sap.0000185460.31188.c1

[23] Agrawal A Sibbering D Courtney CA Skin sparing mastectomy and immediate breast reconstruction: a review. Eur J Surg Oncol. 2013;39:320–328. 23333068 10.1016/j.ejso.2012.12.015

[24] Donker M Hage J Woerdeman L Rutgers EJ Sonke G Vrancken Peeters MJ Surgical complications of skin sparing mastectomy and immediate prosthetic reconstruction after neoadjuvant chemotherapy for invasive breast cancer. Eur J Surg Oncol. 2012;38:25–30. 21963981 10.1016/j.ejso.2011.09.005

[25] Lambert K Mokbel K Does post-mastectomy radiotherapy represent a contraindication to skin-sparing mastectomy and immediate reconstruction: an update. Surg Oncol. 2012;21:e67–e74. 22296996 10.1016/j.suronc.2011.12.007

[26] Lardi AM Ho-Asjoe M Junge K Farhadi J Capsular contracture in implant based breast reconstruction – the effect of porcine acellular dermal matrix. Gland Surg. 2017;6:49–56. 28210552 10.21037/gs.2017.01.02 PMC5293652

[27] Drucker-Zertuche M Robles-Vidal C A 7 year experience with immediate breast reconstruction after skin sparing mastectomy for cancer. Eur J Surg Oncol. 2007;33:140–146. 17112698 10.1016/j.ejso.2006.10.010

[28] Wise MW Hilaire HS Sadeghi A Dupin C Autologous breast reconstruction. In: Riker A, ed. Breast Disease. New York: Springer; 2015: 279–304.

[29] DeLong MR Tandon VJ Rudkin GH Da Lio AL Latissimus dorsi flap breast reconstruction – a nationwide inpatient sample review. Ann Plast Surg. 2017;78:S185–S188. 28346311 10.1097/SAP.0000000000001079

[30] Leff DR Bottle A Mayer E et al Trends in immediate postmastectomy breast reconstruction in the United Kingdom. Plast Reconstr Surg Glob Open. 2015;3:e507. 26495220 10.1097/GOX.0000000000000484 PMC4596432

[31] Pien I Caccavale S Cheung MC et al Evolving trends in autologous breast reconstruction: is the deep inferior epigastric artery perforator flap taking over? Ann Plast Surg. 2016;76:489–493. 25180959 10.1097/SAP.0000000000000339

[32] Schmauss D Machens HG Harder Y Breast reconstruction after mastectomy. Front Surg. 2015;2:71. 26835456 10.3389/fsurg.2015.00071 PMC4717291

[33] Critchley A Thompson A Chan H Reed M Current controversies in breast cancer surgery. Clin Oncol. 2013;25:101–108. 10.1016/j.clon.2012.10.009 23183307

[34] Lee KT Mun GH A systematic review of functional donor-site morbidity after latissimus dorsi muscle transfer. Plast Reconstr Surg. 2014;134:303–314. 24732650 10.1097/PRS.0000000000000365

[35] Lee J Bae Y Jung JH et al Effects of quilting suture interval on donor site seromas after breast reconstruction with latissimus dorsi muscle flap: a randomized trial. Clin Breast Cancer. 2016;16:e159–e164. 27364307 10.1016/j.clbc.2016.05.017

[36] Platt J Baxter N Zhong T Breast reconstruction after mastectomy for breast cancer. Can Med Assoc J. 2011;183:2109–2116. 22065359 10.1503/cmaj.110513 PMC3255143

[37] Nahabedian MY Momen B Galdino G Manson PN Namnoum JD Breast reconstruction with the free TRAM or DIEP flap: patient selection, choice of flap, and outcome. Plast Reconstr Surg. 2002;110:466–475. 12142662 10.1097/00006534-200208000-00015

[38] Sterodimas A de Faria J Nicaretta B Pitanguy I Tissue engineering with adipose-derived stem cells (ADSCs): current and future applications. J Plast Reconstr Aesthet Surg. 2010;63:1886–1892. 19969517 10.1016/j.bjps.2009.10.028

[39] Krumboeck A Giovanoli P Plock JA Fat grafting and stem cell enhanced fat grafting to the breast under oncological aspects – recommendations for patient selection. Breast. 2013;22:579–584. 23769661 10.1016/j.breast.2013.05.006

[40] Locke M Windsor J Dunbar P Human adipose-derived stem cells: isolation, characterization and applications in surgery. ANZ J Surg. 2009;79:235–244. 19432707 10.1111/j.1445-2197.2009.04852.x

[41] Dominici M Le Blanc K Mueller I et al Minimal criteria for defining multipotent mesenchymal stromal cells. The International Society for Cellular Therapy position statement. Cytotherapy. 2006;8:315–317. 16923606 10.1080/14653240600855905

[42] Zuk PA Zhu M Mizuno H et al Multilineage cells from human adipose tissue: implications for cell-based therapies. Tissue Eng. 2001;7:211–228. 11304456 10.1089/107632701300062859

[43] Song L Young NJ Webb NE Tuan RS Origin and characterization of multipotential mesenchymal stem cells derived from adult human trabecular bone. Stem Cells Dev. 2005;14:712–721. 16433626 10.1089/scd.2005.14.712

[44] Choi YS Noh SE Lim SM et al Multipotency and growth characteristic of periosteum-derived progenitor cells for chondrogenic, osteogenic, and adipogenic differentiation. Biotechnol Lett. 2008;30:593–601. 17985079 10.1007/s10529-007-9584-2

[45] De Bari C Dell’Accio F Tylzanowski P Luyten FP Multipotent mesenchymal stem cells from adult human synovial membrane. Arthritis Rheum. 2001;44:1928–1942. 11508446 10.1002/1529-0131(200108)44:8<1928::AID-ART331>3.0.CO;2-P

[46] Dodson MV Hausman GJ Guan L et al Skeletal muscle stem cells fromanimals I. Basic cell biology. Int J Biol Sci. 2010;6:465–474. 20827399 10.7150/ijbs.6.465 PMC2935669

[47] Waked K Colle J Doornaert M Cocquyt V Blondeel P Systematic review: The oncological safety of adipose fat transfer after breast cancer surgery. The Breast. 2 28, 2017;31:128–136. 27837706 10.1016/j.breast.2016.11.001

[48] Miura M Gronthos S Zhao M et al SHED: stem cells from human exfoliated deciduous teeth. Proc Natl Acad Sci U S A. 2003;100:5807–5812. 12716973 10.1073/pnas.0937635100 PMC156282

[49] Seo BM Miura M Gronthos S et al Investigation of multipotent postnatal stem cells from human periodontal ligament. Lancet. 2004;364:149–155. 10.1016/S0140-6736(04)16627-0 15246727

[50] Hass R Kasper C Böhm S Jacobs R Different populations and sources of human mesenchymal stem cells (MSC): a comparison of adult and neonatal tissue-derived MSC. Cell Commun Signal. 2011;9:1. 21569606 10.1186/1478-811X-9-12 PMC3117820

[51] Chatterjee S Laliberte M Blelloch S et al Adipose-derived stromal vascular fraction differentially expands breast progenitors in tissue adjacent to tumors compared to healthy breast tissue. Plast Reconstr Surg. 2015;136:414e-425e. 10.1097/PRS.0000000000001635 PMC4890821 26090768

[52] Bourin P Bunnell BA Casteilla L et al Stromal cells from the adipose tissue-derived stromal vascular fraction and culture expanded adipose tissue-derived stromal/stem cells: a joint statement of the International Federation for Adipose Therapeutics and Science (IFATS) and the International Society for Cellular Therapy (ISCT). Cytotherapy. 2013;15:641–648. 23570660 10.1016/j.jcyt.2013.02.006 PMC3979435

[53] Martin-Padura I Gregato G Marighetti P et al The white adipose tissue used in lipotransfer procedures is a rich reservoir of CD34+ progenitors able to promote cancer progression. Cancer Res. 2012;72:325–334. 22052460 10.1158/0008-5472.CAN-11-1739

[54] Combellack EJ Jessop ZM Naderi N et al Adipose regeneration and implications for breast reconstruction: update and the future. Gland Surg. 2016;5:227–241. 27047789 10.3978/j.issn.2227-684X.2016.01.01 PMC4791352

[55] Brayfield C Marra KG Rubin JP Adipose tissue regeneration. Curr Stem Cell Res Ther. 2010;5:116–121. 19941458 10.2174/157488810791268582

[56] Estes BT Diekman BO Gimble JM Guilak F Isolation of adipose-derived stem cells and their induction to a chondrogenic phenotype. Nat Protoc. 2010;5:1294–1311. 20595958 10.1038/nprot.2010.81 PMC3219531

[57] Lee WC Sepulveda JL Rubin JP Marra KG Cardiomyogenic differentiation potential of human adipose precursor cells. Int J Cardiol. 2009;133:399–401. 18201779 10.1016/j.ijcard.2007.11.068

[58] Uysal AC Mizuno H Tendon regeneration and repair with adipose derived stem cells. Curr Stem Cell Res Ther. 2010;5:161–167. 19941450 10.2174/157488810791268609

[59] Zhang Y Bellows CF Kolonin MG Adipose tissue-derived progenitor cells and cancer. World J Stem Cells. 2010;2:103–113. 21607127 10.4252/wjsc.v2.i5.103 PMC3097931

[60] Niesler CU Siddle K Prins JB Human preadipocytes display a depot-specific susceptibility to apoptosis. Diabetes. 1998;47:1365–1368. 9703343 10.2337/diab.47.8.1365

[61] Tchkonia T Giorgadze N Pirtskhalava T et al Fat depot origin affects adipogenesis in primary cultured and cloned human preadipocytes. Am J Physiol Regul Integr Comp Physiol. 2002;282:R1286–R1296. 11959668 10.1152/ajpregu.00653.2001

[62] Schipper BM Marra KG Zhang W Donnenberg AD Rubin JP Regional anatomic and age effects on cell function of human adipose-derived stem cells. Ann Plast Surg. 2008;60:538–544. 18434829 10.1097/SAP.0b013e3181723bbe PMC4160894

[63] Gimble JM Katz AJ Bunnell BA Adipose-derived stem cells for regenerative medicine. Circ Res. 2007;100:1249–1260. 17495232 10.1161/01.RES.0000265074.83288.09 PMC5679280

[64] Kolonin MG Evans KW Mani SA Gomer RH Alternative origins of stroma in normal organs and disease. Stem Cell Res. 2012;8:312–323. 22209011 10.1016/j.scr.2011.11.005 PMC3571702

[65] Yoshimura K Shigeura T Matsumoto D et al Characterization of freshly isolated and cultured cells derived from the fatty and fluid portions of liposuction aspirates. J Cell Physiol. 2006;208:64–76. 16557516 10.1002/jcp.20636

[66] Tang W Zeve D Suh JM et al White fat progenitor cells reside in the adipose vasculature. Science. 2008;322:583–586. 18801968 10.1126/science.1156232 PMC2597101

[67] Yarak S Okamoto OK Human adipose-derived stem cells: current challenges and clinical perspectives. An Bras Dermatol. 2010;85:647–656. 21152789 10.1590/s0365-05962010000500008

[68] Illouz YG Sterodimas A Autologous fat transplantation to the breast: a personal technique with 25 years of experience. Aesthetic Plast Surg. 2009;33:706–715. 19495856 10.1007/s00266-009-9377-1

[69] Zheng DN Li QF Lei H et al Autologous fat grafting to the breast for cosmetic enhancement: experience in 66 patients with long-term follow up. J Plast Reconstr Aesthet Surg. 2008;61:792–798. 18321802 10.1016/j.bjps.2007.08.036

[70] Khouri RK Eisenmann-Klein M Cardoso E et al Brava and autologous fat transfer is a safe and effective breast augmentation alternative: results of a 6-year, 81-patient, prospective multicenter study. Plast Reconstr Surg. 2012;129:1173–1187. 22261565 10.1097/PRS.0b013e31824a2db6

[71] de Blacam C Momoh AO Colakoglu S Tobias AM Lee BT Evaluation of clinical outcomes and aesthetic results after autologous fat grafting for contour deformities of the reconstructed breast. Plast Reconstr Surg. 2011;128:411e-418e. 10.1097/PRS.0b013e31822b669f 22030501

[72] Choi M Small K Levovitz C Lee C Fadl A Karp NS The volumetric analysis of fat graft survival in breast reconstruction. Plast Reconstr Surg. 2013;131:185–191. 23076412 10.1097/PRS.0b013e3182789b13

[73] Serra-Renom JM Muñoz-Olmo JL Serra-Mestre JM Fat grafting in postmastectomy breast reconstruction with expanders and prostheses in patients who have received radiotherapy: formation of new subcutaneous tissue. Plast Reconstr Surg. 2010;125:12–18. 20048576 10.1097/PRS.0b013e3181c49458

[74] Salgarello M Visconti G Barone-Adesi L Fat grafting and breast reconstruction with implant: another option for irradiated breast cancer patients. Plast Reconstr Surg. 2012;129:317–329. 21987041 10.1097/PRS.0b013e31822b6619

[75] Agha RA Goodacre T Orgill DP Use of autologous fat grafting for reconstruction postmastectomy and breast conserving surgery: a systematic review protocol. BMJ Open. 2013;3:e003709. 10.1136/bmjopen-2013-003709 PMC3808755 24154518

[76] Sinna R Delay E Garson S Delaporte T Toussoun G Breast fat grafting (lipomodelling) after extended latissimus dorsi flap breast reconstruction: a preliminary report of 200 consecutive cases. J Plast Reconstr Aesthet Surg. 2010;63:1769–1777. 20079699 10.1016/j.bjps.2009.12.002

[77] Delay E Garson S Tousson G Sinna R Fat injection to the breast. technique, results, and indications based on 880 procedures over 10 years. Aesthet Surg J. 2009;29:360–376. 19825464 10.1016/j.asj.2009.08.010

[78] Khouri R Del Vecchio D Breast reconstruction and augmentation using pre-expansion and autologous fat transplantation. Clin Plast Surg. 2009;36:269–280. 19309653 10.1016/j.cps.2008.11.009

[79] Missana M Laurent I Barreau L Balleyguier C Autologous fat transfer in reconstructive breast surgery: indications, technique and results. Eur J Surg Oncol. 2007;33:685–690. 17241760 10.1016/j.ejso.2006.12.002

[80] Matsumoto D Sato K Gonda K et al Cell-assisted lipotransfer: supportive use of human adipose-derived cells for soft tissue augmentation with lipoinjection. Tissue Eng. 2006;12:3375–3382. 17518674 10.1089/ten.2006.12.3375

[81] Kølle SF Fischer-Nielsen A Mathiasen AB et al Enrichment of autologous fat grafts with ex-vivo expanded adipose tissue-derived stem cells for graft survival: a randomised placebo-controlled trial. Lancet. 2013;382:1113–1120. 24075051 10.1016/S0140-6736(13)61410-5

[82] Yoshimura K Sato K Aoi N Kurita M Hirohi T Harii K Cell-assisted lipotransfer for cosmetic breast augmentation: supportive use of adipose-derived stem/stromal cells. Aesthetic Plast Surg. 2008;32:48–55. 17763894 10.1007/s00266-007-9019-4 PMC2175019

[83] Vermette M Trottier V Ménard V Saint-Pierre L Roy A Fradette J Production of a new tissue-engineered adipose substitute from human adipose-derived stromal cells. Biomaterials. 2007;28:2850–2860. 17374391 10.1016/j.biomaterials.2007.02.030

[84] Chang Q Lu F A novel strategy for creating a large amount of engineered fat tissue with an axial vascular pedicle and a prefabricated scaffold. Med Hypotheses. 2012;79:267–270. 22688400 10.1016/j.mehy.2012.05.007

[85] Dai R Wang Z Samanipour R Koo KI Kim K Adipose-derived stem cells for tissue engineering and regenerative medicine applications. Stem Cells Int. 2016;2016:6737345. 27057174 10.1155/2016/6737345 PMC4761677

[86] Tajbakhsh S Hajiali F A comprehensive study on the fabrication and properties of biocomposites of poly(lactic acid)/ceramics for bone tissue engineering. Mater Sci Eng C Mater Biol Appl. 2017;70:897–912. 27770967 10.1016/j.msec.2016.09.008

[87] von Heimburg D Zachariah S Kühling H et al Human preadipocytes seeded on freeze-dried collagen scaffolds investigated in vitro and in vivo. Biomaterials. 2001;22:429–438. 11214753 10.1016/s0142-9612(00)00186-1

[88] von Heimburg D Zachariah S Low A Pallua N Influence of different biodegradable carriers on the in vivo behavior of human adipose precursor cells. Plast Reconstr Surg. 2001;108:411–420; discussion 421–422. 11496183 10.1097/00006534-200108000-00020

[89] Halbleib M Skurk T de Luca C von Heimburg D Hauner H Tissue engineering of white adipose tissue using hyaluronic acid-based scaffolds. I: in vitro differentiation of human adipocyte precursor cells on scaffolds. Biomaterials. 2003;24:3125–3132. 12895585 10.1016/s0142-9612(03)00156-x

[90] Hemmrich K von Heimburg D Rendchen R Di Bartolo C Milella E Pallua N Implantation of preadipocyte-loaded hyaluronic acid-based scaffolds into nude mice to evaluate potential for soft tissue engineering. Biomaterials. 2005;26:7025–7037. 15964623 10.1016/j.biomaterials.2005.04.065

[91] Tsuji W Inamoto T Ito R Morimoto N Tabata Y Toi M Simple and longstanding adipose tissue engineering in rabbits. J Artif Organs. 2013;16:110–114. 23114565 10.1007/s10047-012-0670-4

[92] Wu X Black L Santacana-Laffitte G Patrick CW Preparation and assessment of glutaraldehyde-crosslinked collagen-chitosan hydrogels for adipose tissue engineering. J Biomed Mater Res A. 2007;81:59–65. 17109417 10.1002/jbm.a.31003

[93] Morimoto N Yoshimura K Niimi M et al Novel collagen/gelatin scaffold with sustained release of basic fibroblast growth factor: clinical trial for chronic skin ulcers. Tissue Eng Part A. 2013;19(17-18):1931–1940. 23541061 10.1089/ten.tea.2012.0634

[94] Stillaert F Di Bartolo C Hunt J et al Human clinical experience with adipose precursor cells seeded on hyaluronic acid-based spongy scaffolds. Biomaterials. 2008;29:3953–3959. 18635258 10.1016/j.biomaterials.2008.06.005

[95] Tan H Ramirez CM Miljkovic N Li H Rubin JP Marra KG Thermosensitive injectable hyaluronic acid hydrogel for adipose tissue engineering. Biomaterials. 2009;30:6844–6853. 19783043 10.1016/j.biomaterials.2009.08.058 PMC2783716

[96] Hanken H Goehler F Smeets R et al Attachment, viability and adipodifferentiation of pre-adipose cells on silk scaffolds with and without co-expressed FGF-2 and VEGF. In Vivo. 2016;30:567–572. 27566073

[97] Frazier TP Bowles A Lee S et al Serially transplanted nonpericytic CD146(−) adipose stromal/stem cells in silk bioscaffolds regenerate adipose tissue in vivo. Stem Cells. 2016;34:1097–1111. 26865460 10.1002/stem.2325 PMC5886026

[98] Abbott RD Kimmerling EP Cairns DM Kaplan DL Silk as a biomaterial to support long-term three-dimensional tissue cultures. ACS Appl Mater Interfaces. 2016;8:21861–21868. 26849288 10.1021/acsami.5b12114

[99] Mauney JR Nguyen T Gillen K Kirker-Head C Gimble JM Kaplan DL Engineering adipose-like tissue in vitro and in vivo utilizing human bone marrow and adipose-derived mesenchymal stem cells with silk fibroin 3D scaffolds. Biomaterials. 2007;28:5280–5290. 17765303 10.1016/j.biomaterials.2007.08.017 PMC2695965

[100] Fine NA Lehfeldt M Gross JE et al SERI surgical scaffold, prospective clinical trial of a silk-derived biological scaffold in two-stage breast reconstruction: 1-year data. Plast Reconstr Surg. 2015;135:339–351. 25502862 10.1097/PRS.0000000000000987

[101] Clemens MW Downey S Agullo F et al Clinical application of a silk fibroin protein biologic scaffold for abdominal wall fascial reinforcement. Plast Reconstr Surg Glob Open. 2014;2:e246. 25506529 10.1097/GOX.0000000000000217 PMC4255889

[102] Mashhadikhan M Soleimani M Parivar K Yaghmaei P ADSCs on PLLA/PCL hybrid nanoscaffold and gelatin modification: cytocompatibility and mechanical properties. Avicenna J Med Biotechnol. 2015;7:32–38. 25926950 PMC4388888

[103] Huber B Borchers K Tovar GE Kluger PJ Methacrylated gelatin and mature adipocytes are promising components for adipose tissue engineering. J Biomater Appl. 2016;30:699–710. 26017717 10.1177/0885328215587450

[104] Hoch E Hirth T Tovar GE Borchers K Chemical tailoring of gelatin to adjust its chemical and physical properties for functional bioprinting. J Mater Chem B. 2013;1:5675–5685. 10.1039/c3tb20745e 32261191

[105] Hong L Peptan I Clark P Mao JJ Ex vivo adipose tissue engineering by human marrow stromal cell seeded gelatin sponge. Ann Biomed Eng. 2005;33:511–517. 15909657 10.1007/s10439-005-2510-7

[106] Halberstadt C Austin C Rowley J et al A hydrogel material for plastic and reconstructive applications injected into the subcutaneous space of a sheep. Tissue Eng. 2002;8:309–319. 12031119 10.1089/107632702753725067

[107] Jia J Richards DJ Pollard S et al Engineering alginate as bioink for bioprinting. Acta Biomater. 2014;10:4323–4331. 24998183 10.1016/j.actbio.2014.06.034 PMC4350909

[108] Gruene M Pflaum M Deiwick A et al Adipogenic differentiation of laser-printed 3D tissue grafts consisting of human adipose-derived stem cells. Biofabrication. 2011;3:015005. 21358040 10.1088/1758-5082/3/1/015005

[109] Ruvinov E Cohen S Alginate biomaterial for the treatment of myocardial infarction: progress, translational strategies, and clinical outlook: from ocean algae to patient bedside. Adv Drug Deliv Rev. 2016;96:54–76. 25962984 10.1016/j.addr.2015.04.021

[110] Patrick CW Jr. Breast tissue engineering. Annu Rev Biomed Eng. 2004;6:109–130. 15255764 10.1146/annurev.bioeng.6.040803.140032

[111] Patrick C Jr Zheng B Johnston C Reece G Long-term implantation of preadipocyte-seeded PLGA scaffolds. Tissue Eng. 2002;8:283–293. 12031117 10.1089/107632702753725049

[112] Patrick C Jr Chauvin P Hobley J Reece G Preadipocyte seeded PLGA scaffolds for adipose tissue engineering. Tissue Eng. 1999;5:139–151. 10358221 10.1089/ten.1999.5.139

[113] Chhaya MP Melchels FP Holzapfel BM Baldwin JG Hutmacher DW Sustained regeneration of high-volume adipose tissue for breast reconstruction using computer aided design and biomanufacturing. Biomaterials. 2015;52:551–560. 25818460 10.1016/j.biomaterials.2015.01.025

[114] Findlay MW Dolderer JH Trost N et al Tissue-engineered breast reconstruction: bridging the gap toward large-volume tissue engineering in humans. Plast Reconstr Surg. 2011;128:1206–1215. 22094739 10.1097/PRS.0b013e318230c5b2

[115] Choi YS Cha SM Lee YY Kwon SW Park CJ Kim M Adipogenic differentiation of adipose tissue derived adult stem cells in nude mouse. Biochem Biophys Res Commun. 2006;345:631–637. 16696950 10.1016/j.bbrc.2006.04.128

[116] Mironov AV Grigoryev AM Krotova LI Skaletsky NN Popov VK Sevastianov VI 3D printing of PLGA scaffolds for tissue engineering. J Biomed Mater Res A. 2017;105:104–109. 27543196 10.1002/jbm.a.35871

[117] Choi YS Park SN Suh H Adipose tissue engineering using mesenchymal stem cells attached to injectable PLGA spheres. Biomaterials. 2005;26:5855–5863. 15949551 10.1016/j.biomaterials.2005.02.022

[118] Zhang K Song L Wang J et al Strategy for constructing vascularized adipose units in poly (l-glutamic acid) hydrogel porous scaffold through inducing in-situ formation of ASCs spheroids. Acta Biomater. 2017;51:246–257. 28093366 10.1016/j.actbio.2017.01.043

[119] Wiggenhauser PS Müller DF Melchels FP et al Engineering of vascularized adipose constructs. Cell Tissue Res. 2012;347:747–757. 21850493 10.1007/s00441-011-1226-2

[120] Lee TJ Bhang SH La WG et al Volume-stable adipose tissue formation by implantation of human adipose-derived stromal cells using solid free-form fabrication-based polymer scaffolds. Ann Plast Surg. 2013;70:98–102. 22274147 10.1097/SAP.0b013e31822f9a81

[121] Luo L He Y Chang Q et al Polycaprolactone nanofibrous mesh reduces foreign body reaction and induces adipose flap expansion in tissue engineering chamber. Int J Nanomedicine. 2016;11:6471–6483. 27980405 10.2147/IJN.S114295 PMC5147407

[122] Chhaya MP Balmayor ER Hutmacher DW Schantz J-T Transformation of breast reconstruction via additive biomanufacturing. Sci Rep. 2016;6:28030. 27301425 10.1038/srep28030 PMC4908382

[123] Wittmann K Storck K Muhr C et al Development of volume-stable adipose tissue constructs using polycaprolactone-based polyurethane scaffolds and fibrin hydrogels. J Tissue Eng Regen Med. 2013;10:E409–E418. 24170732 10.1002/term.1830

[124] Gugerell A Kober J Laube T et al Electrospun poly (ester-urethane)-and poly (ester-urethane-urea) fleeces as promising tissue engineering scaffolds for adipose-derived stem cells. PLoS ONE. 2014;9:e90676. 24594923 10.1371/journal.pone.0090676 PMC3942471

[125] Lin SD Wang KH Kao AP Engineered adipose tissue of predefined shape and dimensions from human adipose-derived mesenchymal stem cells. Tissue Eng Part A. 2008;14:571–581. 18361763 10.1089/tea.2007.0192

[126] Brandl FP Seitz AK Teßmar JK Blunk T Göpferich AM Enzymatically degradable poly (ethylene glycol) based hydrogels for adipose tissue engineering. Biomaterials. 2010;31:3957–3966. 20170951 10.1016/j.biomaterials.2010.01.128

[127] Pati F Jang J Ha DH et al Printing three-dimensional tissue analogues with decellularized extracellular matrix bioink. Nat Commun. 2014;5:3935. 24887553 10.1038/ncomms4935 PMC4059935

[128] Han TTY Toutounji S Amsden BG Flynn LE Adipose-derived stromal cells mediate in vivo adipogenesis, angiogenesis and inflammation in decellularized adipose tissue bioscaffolds. Biomaterials. 2015;72:125–137. 26360790 10.1016/j.biomaterials.2015.08.053

[129] Wang L Johnson JA Zhang Q Beahm EK Combining decellularized human adipose tissue extracellular matrix and adipose-derived stem cells for adipose tissue engineering. Acta Biomater. 2013;9:8921–8931. 23816649 10.1016/j.actbio.2013.06.035 PMC3965366

[130] Kim JS Choi JS Cho YW Cell-free hydrogel system based on a tissue-specific extracellular matrix for in situ adipose tissue regeneration. ACS Appl Mater Interfaces. 2017;9:8581–8588. 28233976 10.1021/acsami.6b16783

[131] Tan QW Zhang Y Luo JC et al Hydrogel derived from decellularized porcine adipose tissue as a promising biomaterial for soft tissue augmentation. J Biomed Mater Res A. 2017;105:1756–1764. 28165664 10.1002/jbm.a.36025

[132] Pati F Ha D-H Jang J Han HH Rhie J-W Cho D-W Biomimetic 3D tissue printing for soft tissue regeneration. Biomaterials. 2015;62:164–175. 26056727 10.1016/j.biomaterials.2015.05.043

[133] Chan EC Kuo S-M Kong AM et al Three dimensional collagen scaffold promotes intrinsic vascularisation for tissue engineering applications. PLoS ONE. 2016;11:e0149799. 26900837 10.1371/journal.pone.0149799 PMC4762944

[134] Song YH Shon SH Shan M Stroock AD Fischbach C Adipose-derived stem cells increase angiogenesis through matrix metalloproteinase-dependent collagen remodeling. Integr Biol. 2016;8:205–215. 10.1039/c5ib00277j PMC4755818 26758423

[135] Rohringer S Hofbauer P Schneider KH et al Mechanisms of vasculogenesis in 3D fibrin matrices mediated by the interaction of adipose-derived stem cells and endothelial cells. Angiogenesis. 2014;17:921–933. 25086616 10.1007/s10456-014-9439-0

[136] Holnthoner W Hohenegger K Husa AM et al Adipose-derived stem cells induce vascular tube formation of outgrowth endothelial cells in a fibrin matrix. J Tissue Eng Regen Med. 2015;9:127–136. 23038666 10.1002/term.1620

[137] Zhang Q Hubenak J Iyyanki T et al Engineering vascularized soft tissue flaps in an animal model using human adipose–derived stem cells and VEGF+ PLGA/PEG microspheres on a collagen-chitosan scaffold with a flow-through vascular pedicle. Biomaterials. 2015;73:198–213. 26410787 10.1016/j.biomaterials.2015.09.024 PMC4605897

[138] Doldere JH Thompson EW Slavin J et al Long-term stability of adipose tissue generated from a vascularized pedicled fat flap inside a chamber. Plast Reconstr Surg. 2011;127:2283–2292. 21617462 10.1097/PRS.0b013e3182131c3e

[139] Carter JC Church FC Mature breast adipocytes promote breast cancer cell motility. Exp Mol Pathol. 2012;92:312–317. 22445926 10.1016/j.yexmp.2012.03.005

[140] Bertolini F Petit J Kolonin M Stem cells from adipose tissue and breast cancer: hype, risks and hope. Br J Cancer. 2015;112:419–423. 25584493 10.1038/bjc.2014.657 PMC4453662

[141] Dirat B Bochet L Dabek M et al Cancer-associated adipocytes exhibit an activated phenotype and contribute to breast cancer invasion. Cancer Res. 2011;71:2455–2465. 21459803 10.1158/0008-5472.CAN-10-3323

[142] Zhang Y Daquinag AC Amaya-Manzanares F Sirin O Tseng C Kolonin MG Stromal progenitor cells from endogenous adipose tissue contribute to pericytes and adipocytes that populate the tumor microenvironment. Cancer Res. 2012;72:5198–5208. 23071132 10.1158/0008-5472.CAN-12-0294

[143] Zhang Y Daquinag A Traktuev DO et al White adipose tissue cells are recruited by experimental tumors and promote cancer progression in mouse models. Cancer Res. 2009;69:5259–5266. 19491274 10.1158/0008-5472.CAN-08-3444 PMC3857703

[144] Altman AM Prantl L Muehlberg FL et al Wound microenvironment sequesters adipose-derived stem cells in a murine model of reconstructive surgery in the setting of concurrent distant malignancy. Plast Reconstr Surg. 2011;127:1467–1477. 21460655 10.1097/PRS.0b013e31820a6400

[145] Zhao M Dumur CI Holt SE Beckman MJ Elmore LW Multipotent adipose stromal cells and breast cancer development: think globally, act locally. Mol Carcinog. 2010;49:923–927. 20842668 10.1002/mc.20675 PMC3276248

[146] Chandler E Saunders M Yoon C Gourdon D Fischbach C Adipose progenitor cells increase fibronectin matrix strain and unfolding in breast tumors. Phys Biol. 2011;8:015008. 21301062 10.1088/1478-3975/8/1/015008

[147] Ghajar CM Bissell MJ Extracellular matrix control of mammary gland morphogenesis and tumorigenesis: insights from imaging. Histochem Cell Biol. 2008;130:1105–1118. 19009245 10.1007/s00418-008-0537-1 PMC2949356

[148] Nelson CM Bissell MJ Of extracellular matrix, scaffolds, and signaling: tissue architecture regulates development, homeostasis, and cancer. Annu Rev Cell Dev Biol. 2006;22:287–309. 16824016 10.1146/annurev.cellbio.22.010305.104315 PMC2933192

[149] Kass L Erler JT Dembo M Weaver VM Mammary epithelial cell: influence of extracellular matrix composition and organization during development and tumorigenesis. Int J Biochem Cell Biol. 2007;39:1987–1994. 17719831 10.1016/j.biocel.2007.06.025 PMC2658720

[150] Boyd NF Martin LJ Bronskill M Yaffe MJ Duric N Minkin S Breast tissue composition and susceptibility to breast cancer. J Natl Cancer Inst. 2010;102:1224–1237. 20616353 10.1093/jnci/djq239 PMC2923218

[151] Divoux A Clement K Architecture and the extracellular matrix: the still unappreciated components of the adipose tissue. Obes Rev. 2011;12:e494–e503. 21366833 10.1111/j.1467-789X.2010.00811.x

[152] Iyengar P Combs TP Shah SJ et al Adipocyte-secreted factors synergistically promote mammary tumorigenesis through induction of anti-apoptotic transcriptional programs and proto-oncogene stabilization. Oncogene. 2003;22:6408–6423. 14508521 10.1038/sj.onc.1206737

[153] Sasaki Y Miki Y Hirakawa H et al Immunolocalization of estrogen-producing and metabolizing enzymes in benign breast disease: comparison with normal breast and breast carcinoma. Cancer Sci. 2010;101:2286–2292. 20682005 10.1111/j.1349-7006.2010.01673.x PMC11159500

[154] Liu B Ordonez-Ercan D Fan Z et al Estrogenic promotion of ErbB2 tyrosine kinase activity in mammary tumor cells requires activation of ErbB3 signaling. Mol Cancer Res. 2009;7:1882–1892. 19861407 10.1158/1541-7786.MCR-08-0509

[155] Barone I Giordano C Bonofiglio D Andò S Catalano S Leptin, obesity and breast cancer: progress to understanding the molecular connections. Curr Opin Pharmacol. 2016;31:83–89. 27816025 10.1016/j.coph.2016.10.003

[156] Strong AL Strong TA Rhodes LV et al Obesity associated alterations in the biology of adipose stem cells mediate enhanced tumorigenesis by estrogen dependent pathways. Breast Cancer Res. 2013;15:R102. 24176089 10.1186/bcr3569 PMC3978929

[157] Zhang Y Yao F Yao X et al Role of CCL5 in invasion, proliferation and proportion of CD44+/CD24-phenotype of MCF-7 cells and correlation of CCL5 and CCR5 expression with breast cancer progression. Oncol Rep. 2009;21:1113–1121. 19288016

[158] Zimmerlin L Donnenberg AD Rubin JP Basse P Landreneau RJ Donnenberg VS Regenerative therapy and cancer: in vitro and in vivo studies of the interaction between adipose-derived stem cells and breast cancer cells from clinical isolates. Tissue Eng Part A. 2010;17:93–106. 20673000 10.1089/ten.tea.2010.0248 PMC3011910

[159] Donnenberg VS Zimmerlin L Rubin JP Donnenberg AD Regenerative therapy after cancer: what are the risks? Tissue Eng Part B Rev. 2010;16:567–575. 20726819 10.1089/ten.teb.2010.0352 PMC3011999

[160] Cousin B Ravet E Poglio S et al Adult stromal cells derived from human adipose tissue provoke pancreatic cancer cell death both in vitro and in vivo. PLoS ONE. 2009;4:e6278. 19609435 10.1371/journal.pone.0006278 PMC2707007

[161] Ryu H Oh JE Rhee KJ et al Adipose tissue-derived mesenchymal stem cells cultured at high density express IFN-β and suppress the growth of MCF-7 human breast cancer cells. Cancer Lett. 2014;352:220–227. 25016057 10.1016/j.canlet.2014.06.018

[162] Sun B Roh KH Park JR et al Therapeutic potential of mesenchymal stromal cells in a mouse breast cancer metastasis model. Cytotherapy. 2009;11:289–298. 19308770 10.1080/14653240902807026

[163] Krastev T Jonasse Y Kon M Oncological safety of autologous lipoaspirate grafting in breast cancer patients: a systematic review. Ann Surg Oncol. 2013;20:111–119. 22878615 10.1245/s10434-012-2565-2

[164] Zimmerlin L Park TS Zambidis ET Donnenberg VS Donnenberg AD Mesenchymal stem cell secretome and regenerative therapy after cancer. Biochimie. 2013;95:2235–2245. 23747841 10.1016/j.biochi.2013.05.010 PMC3825748

[165] Petit J Botteri E Lohsiriwat V et al Locoregional recurrence risk after lipofilling in breast cancer patients. Ann Oncol. 2012;23:582–588. 21610155 10.1093/annonc/mdr158

[166] Petit J Rietjens M Botteri E et al Evaluation of fat grafting safety in patients with intra epithelial neoplasia: a matched-cohort study. Ann Oncol. 2013;24:1479–1484. 23393126 10.1093/annonc/mds660

[167] Kronowitz SJ Mandujano CC Liu J et al Lipofilling of the breast does not increase the risk of recurrence of breast cancer: a matched controlled study. Plast Reconstr Surg. 2016;137:385–393. 26818270 10.1097/01.prs.0000475741.32563.50

[168] Tan SS Loh W The utility of adipose-derived stem cells and stromal vascular fraction for oncologic soft tissue reconstruction: is it safe? A matter for debate. Surgeon. 2017;15:186–189. 27810224 10.1016/j.surge.2016.09.010

[169] Petit JY Maisonneuve P Rotmensz N et al Safety of lipofilling in patients with breast cancer. Clin Plast Surg. 2015;42:339–344. 26116939 10.1016/j.cps.2015.03.004

[170] Mestak O Hromadkova V Fajfrova M Molitor M Mestak J Evaluation of oncological safety of fat grafting after breast-conserving therapy: a prospective study. Ann Surg Oncol. 2016;23:776–781. 26467459 10.1245/s10434-015-4908-2

[171] Rigotti G Marchi A Stringhini P et al Determining the oncological risk of autologous lipoaspirate grafting for post-mastectomy breast reconstruction. Aesthetic Plast Surg. 2010;34:475–480. 20333521 10.1007/s00266-010-9481-2

[172] Rietjens M De Lorenzi F Rossetto F et al Safety of fat grafting in secondary breast reconstruction after cancer. J Plast Reconstr Aesthet Surg. 2011;64:477–483. 20692216 10.1016/j.bjps.2010.06.024

[173] Petit JY Lohsiriwat V Clough KB et al The oncologic outcome and immediate surgical complications of lipofilling in breast cancer patients: a multicenter study – Milan-Paris-Lyon experience of 646 lipofilling procedures. Plast Reconstr Surg. 2011;128:341–346. 21502905 10.1097/PRS.0b013e31821e713c

[174] Sarfati I Ihrai T Kaufman G Nos C Clough K Adipose-tissue grafting to the post-mastectomy irradiated chest wall: preparing the ground for implant reconstruction. J Plast Reconstr Aesthet Surg. 2011;64:1161–1166. 21514910 10.1016/j.bjps.2011.03.031

[175] Pérez-Cano R Vranckx J Lasso J et al Prospective trial of adipose-derived regenerative cell (ADRC)-enriched fat grafting for partial mastectomy defects: the RESTORE-2 trial. Eur J Surg Oncol. 2012;38:382–389. 22425137 10.1016/j.ejso.2012.02.178

[176] Riggio E Bordoni D Nava MB Oncologic surveillance of breast cancer patients after lipofilling. Aesthetic Plast Surg. 2013;37:728–735. 23812610 10.1007/s00266-013-0166-5

[177] Ihrai T Georgiou C Machiavello JC et al Autologous fat grafting and breast cancer recurrences: retrospective analysis of a series of 100 procedures in 64 patients. J Plast Surg Hand Surg. 2013;47:273–275. 23627644 10.3109/2000656X.2012.759583

[178] Brenelli F Rietjens M De Lorenzi F et al Oncological safety of autologous fat grafting after breast conservative treatment: a prospective evaluation. Breast J. 2014;20:159–165. 24450421 10.1111/tbj.12225

[179] Semprini G Cattin F Zanin C et al About locoregional recurrence risk after lipofilling in breast cancer patients: our experience. Minerva Chir. 2014;69:91–96. 24847895

[180] Gale KL Rakha EA Ball G Tan VK McCulley SJ Macmillan RD A case-controlled study of the oncologic safety of fat grafting. Plast Reconstr Surg. 2015;135:1263–1275. 25919241 10.1097/PRS.0000000000001151

[181] Masia J Bordoni D Pons G Liuzza C Castagnetti F Falco G Oncological safety of breast cancer patients undergoing free-flap reconstruction and lipofilling. Eur J Surg Oncol. 2015;41:612–616. 25800344 10.1016/j.ejso.2015.02.008

[182] Pinell-White XA Etra J Newell M Tuscano D Shin K Losken A Radiographic implications of fat grafting to the reconstructed breast. Breast J. 2015;21:520–525. 26133468 10.1111/tbj.12450

[183] Silva-Vergara C Fontdevila J Descarrega J Burdio F Yoon TS Grande L Oncological outcomes of lipofilling breast reconstruction: 195 consecutive cases and literature review. J Plast Reconstr Aesthet Surg. 2016;69:475–481. 26876108 10.1016/j.bjps.2015.12.029

[184] Kaoutzanis C Xin M Ballard TN et al Autologous fat grafting after breast reconstruction in postmastectomy patients: complications, biopsy rates, and locoregional cancer recurrence rates. Ann Plast Surg. 2016;76:270–275. 26101979 10.1097/SAP.0000000000000561

[185] García RM Alonso VG Doménech MEV Fat grafting in immediate breast reconstruction. Avoiding breast sequelae. Breast Cancer. 2016;23:134–140. 24872086 10.1007/s12282-014-0541-3

[186] De Decker M De Schrijver L Thiessen F Tondu T Van Goethem M Tjalma W Breast cancer and fat grafting: efficacy, safety and complications – a systematic review. Eur J Obstet Gynecol Reprod Biol. 2016;207:100–108. 27835828 10.1016/j.ejogrb.2016.10.032

[187] Al Sufyani MA Al Hargan AH Al Shammari NA Al Sufyani MA Autologous fat transfer for breast augmentation: a review. Dermatol Surg. 2016;42:1235–1242. 27618391 10.1097/DSS.0000000000000791

[188] Spear SL Coles CN Leung BK Gitlin M Parekh M Macarios D The safety, effectiveness, and efficiency of autologous fat grafting in breast surgery. Plast Reconstr Surg Glob Open. 2016;4:e827. 27622095 10.1097/GOX.0000000000000842 PMC5010318

[189] Groen J Negenborn V Twisk D et al Autologous fat grafting in onco-plastic breast reconstruction: a systematic review on oncological and radiological safety, complications, volume retention and patient/surgeon satisfaction. J Plast Reconstr Aesthet Surg. 2016;69:742–764. 27085611 10.1016/j.bjps.2016.03.019

[190] Charvet HJ Orbay H Wong MS Sahar DE The oncologic safety of breast fat grafting and contradictions between basic science and clinical studies: a systematic review of the recent literature. Ann Plast Surg. 2015;75:471–479. 26360655 10.1097/SAP.0000000000000604

[191] Pearl RA Leedham SJ Pacifico MD The safety of autologous fat transfer in breast cancer: lessons from stem cell biology. J Plast Reconstr Aesthet Surg. 2012;65:283–288. 21820375 10.1016/j.bjps.2011.07.017 PMC6485453

[192] Choron RL Chang S Khan S et al Paclitaxel impairs adipose stem cell proliferation and differentiation. J Surg Res. 2015;196:404–415. 25891676 10.1016/j.jss.2015.03.026 PMC4442730

[193] Harris WM Zhang P Plastini M et al Evaluation of function and recovery of adipose-derived stem cells after exposure to paclitaxel. Cytotherapy. 2017;19:211–221. 27887867 10.1016/j.jcyt.2016.10.010

[194] Chen DR Lu DY Lin HY Yeh WL Mesenchymal stem cell-induced doxorubicin resistance in triple negative breast cancer. Biomed Res Int. 2014;2014:532161. 25140317 10.1155/2014/532161 PMC4124237

[195] Kucerova L Skolekova S Matuskova M Bohac M Kozovska Z Altered features and increased chemosensitivity of human breast cancer cells mediated by adipose tissue-derived mesenchymal stromal cells. BMC Cancer. 2013;13:1. 24209831 10.1186/1471-2407-13-535 PMC3829110

[196] Beane OS Fonseca VC Darling EM Adipose-derived stem cells retain their regenerative potential after methotrexate treatment. Exp Cell Res. 2014;327:222–233. 24992046 10.1016/j.yexcr.2014.06.015 PMC4164584

[197] Duong MN Cleret A Matera EL et al Adipose cells promote resistance of breast cancer cells to trastuzumab-mediated antibody-dependent cellular cytotoxicity. Breast Cancer Res. 2015;17:57. 25908175 10.1186/s13058-015-0569-0 PMC4482271

[198] Zheng A Kallio A Harkonen P Tamoxifen-induced rapid death of MCF-7 breast cancer cells is mediated via extracellularly signal-regulated kinase signaling and can be abrogated by estrogen. Endocrinology. 2007;148:2764–2777. 17363451 10.1210/en.2006-1269

[199] Pike S Zhang P Wei Z et al In vitro effects of tamoxifen on adipose-derived stem cells. Wound Repair Regen. 2015;23:728–736. 26043659 10.1111/wrr.12322

[200] Shukla L Morrison WA Shayan R Adipose-derived stem cells in radiotherapy injury: a new frontier. Front Surg. 2015;2:1. 25674565 10.3389/fsurg.2015.00001 PMC4309196

[201] Haubner F Muschter D Pohl F Schreml S Prantl L Gassner HG A co-culture model of fibroblasts and adipose tissue-derived stem cells reveals new insights into impaired wound healing after radiotherapy. Int J Mol Sci. 2015;16:25947–25958. 26528967 10.3390/ijms161125935 PMC4661794

[202] Haubner F Gassner H Potential of adipose-derived stem cells concerning the treatment of wound healing complications after radiotherapy. HNO. 2015;63:111–117. 25630697 10.1007/s00106-014-2953-y

[203] Luan A Duscher D Whittam AJ et al Cell-assisted lipotransfer improves volume retention in irradiated recipient sites and rescues radiation-induced skin changes. Stem Cells. 2016;34:668–673. 26661694 10.1002/stem.2256 PMC4868181

[204] Hasdemir M Agir H Eren GG et al Adipose-derived stem cells improve survival of random pattern cutaneous flaps in radiation damaged skin. J Craniofac Surg. 2015;26:1450–1455. 26114538 10.1097/SCS.0000000000001852

[205] Rigotti G Marchi A Galie M et al Clinical treatment of radiotherapy tissue damage by lipoaspirate transplant: a healing process mediated by adipose-derived adult stem cells. Plast Reconstr Surg. 2007;119:1409–1422. 17415234 10.1097/01.prs.0000256047.47909.71

[206] Zielins ER Brett EA Longaker MT Wan DC Autologous fat grafting: the science behind the surgery. Aesthet Surg J. 2016;36:488–496. 26961989 10.1093/asj/sjw004 PMC5006291

[207] Hivernaud V Lefourn B Guicheux J et al Autologous fat grafting in the breast: critical points and technique improvements. Aesthetic Plast Surg. 2015;39:547–561. 26085223 10.1007/s00266-015-0503-y

